# Quantum Dot-Based Luminescent Sensors: Review from Analytical Perspective

**DOI:** 10.3390/ijms26146674

**Published:** 2025-07-11

**Authors:** Alissa Loskutova, Ansar Seitkali, Dinmukhamed Aliyev, Rostislav Bukasov

**Affiliations:** Department of Chemistry, Nazarbayev University, Kabanbay Batyr Ave. 53, Astana 010000, Kazakhstan

**Keywords:** quantum dots, applications of nanomaterials, LOD, sensitivity, luminescence, chemiluminescence, fluorescence, phosphorescence

## Abstract

Quantum Dots (QDs) are small semiconductor nanoparticles (<10 nm) with strong, relatively stable, and tunable luminescent properties, which are increasingly applied in the sensing and detection of various analytes, including metal ions, biomarkers, explosives, proteins, RNA/DNA fragments, pesticides, drugs, and pollutants. In this review, we critically assess recent developments and advancements in luminescent QD-based sensors from an analytical perspective. We collected, tabulated, and analyzed relevant data reported in 124 peer-reviewed articles. The key analytical figures of merit, including the limit of detection (LOD), excitation and emission wavelengths, and size of the particles were extracted, tabulated, and analyzed with graphical representations. We calculated the geometric mean and median LODs from those tabulated publications. We found the following geometric mean LODs: 38 nM for QD-fluorescent-based sensors, 26 nM for QD-phosphorescent-based sensors, and an impressively low 0.109 pM for QD-chemiluminescent-based sensors, which demonstrate by far the best sensitivity in QD-based detection. Moreover, AI-based sensing methods, including the ATTBeadNet model, optimized principal component analysis(OPCA) model, and Support Vector Machine (SVM)-based system, were reviewed as they enhance the analytical performance of the detection. Despite these advances, there are still challenges that include improvements in recovery values, biocompatibility, stability, and overall performance. This review highlights trends to guide the future design of robust, high-performance, QD-based luminescent sensors.

## 1. Introduction

Quantum dots are nanoparticles with semiconducting properties whose size is, at most, twice as large as the size of the Bohr radius of corresponding exciton and whose electronic properties differ from that of the bulk material they were made of due to a higher surface-to-volume ratio and consequent high reactivity [1,2,3]. They are usually composed of the elements of the groups II A to VI A or III A to V A; however, there are also variations, with IV A–VI A, I B–III A–VI A, or IV A group elements [1,4]. The size of QDs varies from a few nanometers to a few tens of nanometers, which allows for a gradual increase of 1–2 eV in the energy of the electronic transition compared with the bulk material [2,3]. Their unique photophysical characteristics, such as wide absorption spectra, adjustable and small emission profiles, and strong resistance towards photobleaching, allow them to have many applications in biosensing and imaging [5,6]. QDs have discrete energy levels, and by changing their size or alloying their core, one can modulate their bandgap [7,8,9]. QDs also exhibit one of the most important properties of quantum confinement, which makes them useful in the production of light-emitting diodes (LEDs), transistors, and solar cells [3].

Quantum dots have attracted the interest of scientists since the 1980s. They emerged as a result of advancements in chemical processes for production in the doping of multicomponent glasses, the synthesis of nanoparticles by colloidal chemistry, and the epitaxial growth of nanostructures and thin films [10]. In 1981, Alexey Ekimov and Alexey Onushchenko designed the first QDs made of CuCl [11]. Microcrystals grown in the transparent dielectric matrix have demonstrated the short wave shift (up to 0.1 eV) of exciton absorption bands due to the quantum size effect. They have used multicomponent silicate glasses with copper and chlorine added as their compounds in a concentration of about 1%. The treatment of such glasses with high temperatures created the characteristic spectra of the exciton absorption of CuCl crystals in the transparent regions of the matrix, which points out the formation of a solid supersaturated solution due to phase decomposition. The term “quantum dots” was first introduced by M. A. Reed et al. in their 1986 publication about spatial quantization in GaAs–AlGaAs multiple quantum dots [12]. Over the next fifteen years, the quantum size effect in the absorption spectra was studied for quantum dots fabricated from copper halides and cadmium chalcogenides, such as CuCl, CuBr, CdSe, CdS, and CdSSe [13,14,15,16,17,18,19,20].

The most common synthetic methods of synthesizing QDs include colloidal and hydrothermal synthesis. Colloidal synthesis, especially the high-temperature hot injection method, allows for the production of high-quality QDs with controlled size and composition by creating conditions for rapid nucleation and growth [21]. That method results in the high purity, uniformity, and photochemical stability of QDs, which is crucial for optoelectronic applications [3]. Recent advancements, including the use of new ligands and environmentally friendly, aqueous-based methods, have improved control over QD properties [3]. Generally, the aqueous synthesis of QDs requires the presence of three starting components: a metal precursor, which is typically a water-soluble salt; a chalcogenide, such as S, Se, or Te; and a stabilizer [10]. Chalcogenizers, such as NaHTe or NaHSe, are being made in situ from Se or Te powder and NaBH_4_ as a reducing agent; however, sometimes potassium salts of chalcogens, such as K_2_TeO_3_, are being used instead of pure elements [22,23,24]. Stabilizers are meant to provide control over nucleation at early stages and restrict particle growth, which allows precise size control [10]. Additionally, machine learning is increasingly used to optimize synthesis conditions, enhancing efficiency and performance prediction [25,26,27]. The hydrothermal method is a cost-effective and easy approach for synthesizing quantum dots with a controlled size and shape. It involves heating a precursor solution containing metal ions and ligands in a sealed, high-pressure vessel. As the temperature and pressure increase, the solution becomes supersaturated, leading to QD nucleation. Parameters like the temperature, pressure, and reaction time can be adjusted to tune the QD properties. Compared to other techniques, hydrothermal synthesis operates at lower temperatures and is more accessible [3].

Apart from traditional II–VI group QDs, silicon QDs (SiQDs) have gained the attention of researchers due to their simple synthesis, low cost, excellent optical characteristics, low biotoxicity, and environmental friendliness [28]. Reports have been made about the application of SiQDs for the detection of L-cysteine, hydrogen sulfide, vitamin B12, nitrite, hydroquinone, and other types of compounds [29,30,31,32,33]. Another new direction in the development of QDs is focused on perovskite QDs (Per-QDs). Their adjustable redox characteristics, thermal and chemical durability, active electrical structure, electronic and ionic conduction, oxygen sorption capacity, and high oxygen mobility make them solid candidates for optical sensors to detect trace amounts of both organic and inorganic compounds, as well as biomolecules [34]. Among synthetic methods, the sol-gel approach appears to be particularly advantageous for Per-QDs since it allows for precise control over stoichiometry and homogeneity of the final product [35].

In order to stabilize QDs and achieve desirable surface characteristics, scientists utilize ligand engineering. For example, Zhang and coworkers have developed fluorophenethyl ammonium bromide (FPEABr) ligands to modify the surface of CsPbBr_3_ Per-QDs. Their results have shown successful adsorption of ligands onto the QDs’ surface and a decrease in bromine vacancy defects. The application of FPEABr allowed for a strong quantum yield of over 90% and the good stability of the material, which have led to an improved maximum luminance of Per-QD light-emitting diodes (QLEDs) [36]. Jiang et al. have utilized phenethylamine (PEA) to modify the InP QDs used in QLEDs, where some of the long-chain oleylamine ligands were replaced by short-chain PEA ligands. PEA-engineered QDs have exhibited a higher quantum yield (71.0% vs. 85.5%) and a higher maximum external quantum efficiency (1.9% vs. 3.5%) [37].

Machine learning (ML) currently is also widely used to design and adjust the synthetic procedure. It determines the required conditions for synthesizing QDs with desirable properties, thus optimizing the process. In contrast, it is nearly impossible to consider all the possible parameter combinations manually [38]. For example, Chen et al. have demonstrated the use of ML tools to analyze the various conditions required for the preparation of CsPbX3 (where X stands for halogen) Per-QDs. The authors report that ML not only assisted in the elucidation of the nucleation-growth process but also allowed for the synthesis of Per-QDs with an exact emission wavelength and a full width at half the maximum (FWHM) of the photoluminescence spectrum. With ML, they have analyzed different values of parameters such as the Pb:Cs molar ratio, reaction halogen ratio, reaction temperature, and flow rate. As a result, the ML model has satisfactorily predicted the synthesis result, depending on the given conditions. Moreover, the developed tool allows for applying other restrictions apart from the wavelength and the FWHM, including, for instance, the shortest time, lower temperature, or minimum lead amount [38]. Abdel-Latif et al. have utilized AI technology similarly; however, the number of adjustable parameters was expanded to 10, while the accessible parameter space exceeded 2 × 10^7^ [39]. Such opportunities offered by AI development play a crucial role in designing new sensor devices with improved performance.

Before proceeding to our review, we also searched for previously published review studies on the topic of luminescent quantum dot-based sensing. Most of the review papers that we found were published in the last 4 years. Atabaev’s 2018 mini-review of heteroatom-doped carbon dots (CDs) focuses on synthesis methods and their adjustable photoluminescence for sensing and bioimaging applications [40]. Ding et al. discuss the quantum dot–based biosensors for antibiotic residues, detailing QD types (Cd-based, carbon, graphene), functionalization strategies, and optical transduction mechanisms (quenching, energy transfer) in complex samples [41]. Sabzehmeidani et al. provide insights into various QD platforms (metal, carbon, g-C_3_N_4_) and their fluorescent/electrochemical sensing mechanisms to detect antibiotics, evaluating detection metrics across luminescent and electrochemical methods [42]. Mehta and colleagues review electrochemiluminescent (ECL) QDs, discussing synthesis, surface engineering, ECL mechanisms, and their integration into sensitive, low-background analytical devices [43]. Finally, the latest 2023 review by Sargazi et al. offers a broad comparison of fluorescent nanomaterials, not limited to QDs, for antibiotic sensing in food and environmental matrices, emphasizing design principles and fluorescence pathways [44]. Among those reviews published previously, we found no exhaustive comparison of analytical performance parameters, such as the limit of detection (LOD), analytical range, excitation/emission wavelength, relative standard deviation of luminescence intensity signal (RSD), and others. Here we tabulate the results from the papers reporting these parameters, calculate the average and median values for three major luminescence methods, including fluorescence, phosphorescence, and chemiluminescence, and finally discuss the differences between these methods, as well as their strong and weak sides.

## 2. Sensing Applications Based on the Fluorescence of QDs

Fluorescence, along with phosphorescence, is one of the two types of photoluminescence, which can be described as the emission of light resulting from the absorption of electromagnetic radiation, usually in the form of light. For example, when excited by UV radiation, fluorescent material emits light within the visible range. The absorption of a photon with a certain wavelength excites the molecule, making it reach a higher energy level. Then, after a certain time, the molecule relaxes back to the ground state and emits a photon. For fluorescence, this transition occurs from the excited singlet state to the ground singlet state, where the spin of the molecule does not change, and thus the fluorescence lifetime is short (in the ns range) [28].

Quantum dots possess properties different from bulk materials due to quantum confinement and are frequently used as fluorescent nanoprobes. QDs emit fluorescence due to the recombination of electrons and holes (excitons), and this fluorescence can be influenced by modifications to their surface or ligands, which affect their exciton recombination efficiency. By changing these surface characteristics, QDs can be tuned for application in fluorescent sensors that detect target analytes through their either direct or indirect interactions with QDs. The analyte concentration is determined based on the linear relationship between the fluorescence intensity and the analyte concentration. Functional QD-based sensors are typically developed by adjusting surface ligands to enable specific interactions—such as adsorption or chelation—with target molecules [45]. The fluorescent properties of QDs can be tuned by adjusting their size: bigger particles will have a smaller band gap between valence and conduction bands and thus will emit light with longer wavelengths (red shift), while smaller particles appear to emit light with shorter wavelengths (blue shift) due to the larger band gap. The fluorescence lifetime, ability to control the particle size, narrow emission, and wide excitation spectra of QDs allow for their wide application in biosensing, bioimaging, nanomedicine, LED lamps, and solar cells [46,47,48]. One of the first applications of QDs in fluorescence sensor design was demonstrated by Depu Chen’s group in 2001 for the detection of human Immunoglobulin G (IgG) with ZnS-coated CdSe QDs attached to the detection antibody of a sandwich-type immunocomplex with an LOD of 1 nM [49].

There are multiple applications of surface-enhanced fluorescence in biosensing, including in surface-enhanced fluorescence (SEF), that apply QDs and CDs [50]. For instance, there are applications of commercially available QDs and organic CDs in SEF at the surface of bacteria on various substrates including gold, and Al film, which reported high SEF enhancement factors (EF) of up to several 100s and a high contrast of the same order of magnitude up to several 100s [51,52,53]. It was also found that SEF EF had a strong negative correlation with the measured toxicity of QDs, at least for the observation of QD-labeled bacteria, or, in other words, the highest fluorescent enhancement was demonstrated by the least toxic QDs [51]. Therefore, the cytotoxicity of QDs should be minimized for the optimal/effective imaging of QD-labeled bacteria.

Oftentimes, QD-based sensing devices rely on fluorescence quenching strategies such as FRET. Fluorescence (or Förster) resonance energy transfer (FRET) is a quantum mechanical process of resonance between transition dipoles. It requires donor (fluorophore) and acceptor (chromophore) particles, and the fluorescence spectrum of the donor particle should overlap with the absorption spectrum of the acceptor particle. FRET is very sensitive to the distance between particles, which should be kept at 5–10 nm [54]. Their wide absorption and tunable emission bands, which allow for the controllable overlap of emission and absorption spectra, as well as the large Stokes shift to suppress the direct excitation from acceptors, make QDs excellent FRET donors [55].

In addition to FRET, other mechanisms, such as the inner filter effect (IFE), static quenching, and dynamic quenching can also influence fluorescence. IFE leads to a decrease in fluorescence intensity without affecting the fluorophore’s lifetime, since it is created by the absorption of excitation or the emission of light by other sample components. Oppositely, static quenching involves the formation of a non-fluorescent complex between the fluorophore and quencher in the ground state, while dynamic quenching results from collisional interactions in the excited state. Both static and dynamic quenching reduce the fluorescence intensity, but they impact the fluorescence lifetime differently. These quenching mechanisms are often identified by analyzing spectral overlaps and lifetime changes [34].

QDs have attracted a lot of attention due to their outstanding optical properties, including high brightness, photostability, and tunable emission spectra, which make them effective fluorescent probes for sensing devices developed to detect a wide range of analytes in biomedical, environmental, and industrial contexts. However, a systematic comparison of QD-based fluorescence sensors’ performance across different analyte types remains limited in the literature. To fill in this void in QD-related literature, we collected, tabulated, and analyzed data from 42 peer-reviewed analytical papers, just for QD fluorescence-based sensing. The attention was mainly driven to such parameters as the limit of detection (LOD), linear range, and relative standard deviation (RSD). Other parameters, including the excitation and emission wavelength, quantum yield, and recovery percent were also recorded. Table 1 demonstrates the data collected from those papers.

As shown in Table 2, QD-based fluorescence sensors generally exhibit high sensitivity, with most of the values being in the nM range, and the geometric mean and median being equal to 38 nM and 23 nM, respectively. The analysis of the linear range width has shown that currently papers often lack performance according to this parameter since the median is somewhat lower than two orders of magnitude, and the average just slightly exceeds it. Although there were some examples of extremely wide linear ranges spanning 8.00 orders of magnitude, the majority of the values lie within 1.00–3.00 orders of magnitude, which underlines the necessity for improvement.

**Figure 1:**
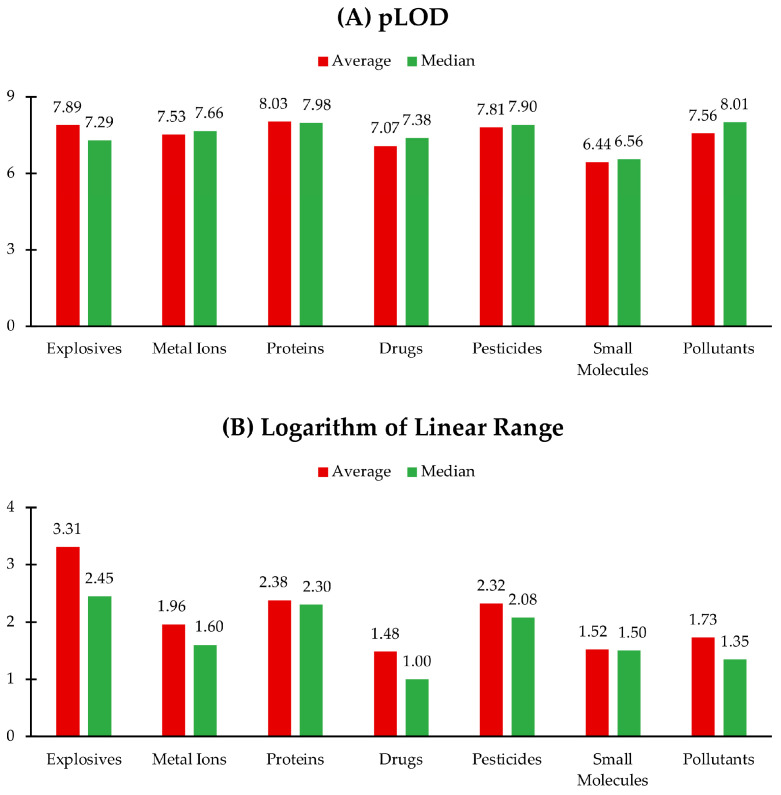
Column charts showing the analytical performance of 42 sensors evaluated based on the (**A**) geometric mean and median of negative logarithm of LOD (pLOD) and (**B**) on the width of the linear range (average and median) for 7 categories of analytes: explosives [57,58,61,78,84], metal ions [23,28,64,69,75,77,83,88], proteins [34,59,63,72], drugs [24,74,80,82,85,86,92], pesticides [73,76,81], small biological molecules [56,65,66,68,70,79,89,91,92], and pollutants [33,60,62,67,71,79,87,90].

All the analytes detected in 42 analytical papers were divided into seven groups: explosives, metal ions, proteins, drugs, pesticides and herbicides, small biological molecules, and environmental and industrial pollutants. Then, the geometric mean and median of the LOD and the average and the median of the logarithmic range were calculated for each group and compared. Figure 1 highlights the areas where QD-based fluorescent sensors perform well, as well as the areas where improvements are awaited.

**Table 1:** Analytical performance in QD-based fluorescent sensors.

First Name	Nanostructure	Size	Sensing Molecule	Excitation and Emission Wavelength	Quenching Mechanism	Analyte	LOD	Range	Log(Range)	RSD (Average, Range)	Other FoM	Preparation
Hong 2012, [56]	CdTe QDs@TGA-Eu^3+^	N/A	N/A	ex.: 360 nm; em.: 540 nm	PET	nucleoside triphosphates	2.0 nM	70–150 nM	0.33	N/A	N/A	reflux synthesis, CdCl_2_, NaHTe, TGA
Ban 2015, [57]	amine-capped Si QDs	2.1 nm	APTMS	ex.: 350 nm; em.: 465 nm	FRET	TNT	1.0 nM	5–500 nM	2.00	2.63%; 2.3–2.9%	QY: 22%	hydrothermal; APTMS, trisodium citrate
Peveler 2015, [58]	red ZnS-shelled CdSe QDs	N/A	OMe	ex.: 365 nm, em.: 608 nm	ET	DNT	0.38 µM	15–85 µM	0.75	N/A	N/A	decomposition; CdO, TOP-Se, Zn-DTCA, surface ligands CD, CX, OH, OMe)
Peveler 2015, [58]	green ZnS-shelled CdSe QDs	N/A	CX	ex.: 365 nm, em.: 544 nm	ET	TNT	0.44 µM	15–85 µM	0.75	N/A	Quenching %: 73%	decomposition; CdO, TOP-Se, Zn-DTCA, surface ligands CD, CX, OH, OMe)
Peveler 2015, [58]	red ZnS-shelled CdSe QDs	N/A	OMe	ex.: 365 nm, em.: 608 nm	ET	tetryl	0.73 µM	15–85 µM	0.75	N/A	Quenching %: 47%	decomposition; CdO, TOP-Se, Zn-DTCA, surface ligands CD, CX, OH, OMe)
Peveler 2015, [58]	red ZnS-shelled CdSe QDs	N/A	OMe	ex.: 365 nm, em.: 608 nm	ET	RDX	2.5 µM	15–85 µM	0.75	N/A	N/A	decomposition; CdO, TOP-Se, Zn-DTCA, surface ligands CD, CX, OH, OMe)
Peveler 2015, [58]	blue ZnS-shelled CdSe QDs	N/A	OH	ex.: 365 nm, em.: 516 nm	ET	PETN	0.35 µM	15–85 µM	0.75	N/A	N/A	decomposition; CdO, TOP-Se, Zn-DTCA, surface ligands CD, CX, OH, OMe)
Zhang 2015, [59]	carboxylated CdT QDs	10 nm	MIPs	ex.: 397 nm, em.: 540 nm	ET	phycocyanin	5.9 nM	0.02–0.8 μM	1.60	3.20%	Recovery %: 94.0–105.0%	hot injection method in aqueous phase; Cd(NO_3_)_2_·4H_2_O, Te powder, NaBH4
Li 2016, [60]	CdTe QDs	3.08 nm	aptamer	ex.: 400 nm, em.: 543 nm	IFE	bisphenol A	8.2 nM	10–80 ng/mL	0.90	1.62%; 1.08–1.91%	Recovery %: 95.3–102%	microwave-assisted; N2-saturated CdCl_2_, Te powder, NaBH_4_, TGA
Qian 2016, [61]	3-MPA-CdTe gQDs@SiO2@ Lcys-CdTe rQDs	3.18 nm	L-cysteine (Lcys)	ex.: 365 nm, em.: 625 nm	static quenching	TNT	3.3 nM	10–8000 nM	2.90	5.28%; 3.5–8.7%	Recovery %: 95.5–108.5%	colloidal; Te powder, NaBH_4_, CdCl_2_
Singh 2016, [62]	ZnO QDs	N/A	N/A	ex.: 320 nm, em.: 525 nm	static and dynamic quenching	free chlorine	41 nM	0.05–0.7 μM	1.15	N/A	N/A	ultrasonication; Zn(OAc)_2_, ethanol, APTES
Chang 2017, [63]	Mn-doped MBA-capped ZnS QDs	3.5 nm	MBA	ex.: 310 nm, em.: 610 nm	no quenching	transferrin (TRF)	5.7 nM	0.1–10 μM	2.00	0.43%; 0.2–0.8%	Recovery %: 86.9–97.5%	wet-chemical precipitation method; ZnSO_4_ × 7H_2_O, MnSO_4_ × 4H_2_O
Qian 2017, [64]	rQDs@SiO2@gQDs	2.34 nm (gQDs); 3.62 nm (rQDs)	1,10-phenanthroline (phen)	ex.: 365 nm, em.: 640 nm	static quenching	Cd^2+^	0.17 nM	0.5–2000 nM	3.60	5.37%; 4.2–6.7%	Recovery %: 96.4–101.4%	colloidal; Te powder, NaBH_4_, CdCl_2_, MPA
Tang 2017, [65]	aN QDs (amino-nitrogen)	5.0 nm	cysteine	ex.: 320 nm, em.: 419 nm	FRET	cysteine	0.10 µM	0.3–3 µM	1.00	2.83%; 1.5–3.7%	QY: 34%; Recovery %: 90.0–106.7%	microwave-assisted; 2-azido imidazole, ammonia
Yu 2017, [66]	Polymer CdTe/CdS QDs	10 nm	CCP	ex.: 380 nm, em.: 420 nm	FRET	hydrogen peroxide	0.10 mM	0.2–4 mM	1.30	N/A	N/A	colloidal; Na_2_TeO_3_, NaBH_4_, CdCl_2_, MPA
Yu 2017, [66]	Polymer CdTe/CdS QDs	10 nm	CCP	ex.: 380 nm, em.: 420 nm	FRET	glucose	50 µM	0.1–5 mM	1.70	N/A	Recovery %: 94.93–105.89%	colloidal; Na_2_TeO_3_, NaBH_4_, CdCl_2_, MPA
Zhou 2017, [67]	Eu-ZnO QDs	5 nm	DPA	ex.: 360 nm, em.: 530 nm	no quenching	CaDPA	3.0 nM	0.004–4 µM	3.00	N/A	QY: 1.89% without DPA, 10.69% with DPA	sol-gel; Zn(OAc)_2_, KOH, APTES, Eu(NO_3_)_3_
Liu 2018, [33]	Si QDs	6.87 nm	N/A	ex.: 410 nm, em.: 512 nm	ET	hydroquinone	2.6 µM	6–100 μM	1.22	2.17%; 1.242–3.464%	Recovery %: 92.5–105.1%	hydrothermal; DAMO
Pourghobadi 2018, [68]	TGA-CdTe QDs	3 nm	TGA	ex.: 360 nm, em.: 580 nm	ET	dopamine	0.35 µM	0.5–10 μM	1.30	4.92%; 2.5–6.28%	Recovery %: 92–106%	aqueous; Te powder, NaBH_4_, CdCl_2_, TGA
Xing 2018, [69]	MAA-ZnTe QDs	12 nm	N/A	ex.: 289 nm, em.: 551 nm	static quenching	Fe^3+^	4.9 µM	2–100 µM	1.70	3.88%	N/A	colloidal; Zn(OAc)_2_, Na_2_TeO_3_, MAA
Zhao 2018, [70]	WS_2_ QDs	8 nm	N/A	ex.: 360 nm, em.: 445 nm	FRET	dopamine	3.3 µM	5–50 μM	1.00	N/A	QY: 21.75%	liquid exfoliation of bulk crystals; WS_2_, 1-methyl-pyrrolidinone
Chen 2019, [23]	CdTe QDs	2.25 nm	TGA	ex.: 330 nm, em.: 602 nm	dynamic quenching	Ag^+^	5.0 nM	5–200 nM	1.60	5.40%; 2.1–9.7%	QY: 56% compared with Rhodamine B in ethanol; Recovery %: 94.5–112.3%	aqueous; K_2_TeO_3_, NaBH_4_, Cd(OAc)_2_, TGA
Feng 2019, [71]	MIPs layer coated on CdTe QDs	N/A	MIPs	ex.: 350 nm, em.: 570 nm	static quenching	tetrabromobisphenol-A	0.55 nM	1.0–60.0 ng/mL	1.78	3.11%; 0.9–6.2%	QY of QDs: 64%; QY of MIP-QDs: 35%; Recovery %: 89.6–107.9%	aqueous; CdCl_2_, K_2_TeO_3_
Li 2019, [72]	SiQDs	2.5 nm	N/A	ex.: 390 nm, em.: 460 nm	IFE	bovine hemoglobin	12 nM	0.01–10 μM	3.00	N/A	QY: 19.47%; Recovery %: 93.7–109.0%	hydrothermal; APTES, TSIM, L-AA
Najafi 2019, [73]	Pd-doped CdTe QDs	3 nm	TGA	ex.: 340 nm, em.: 529 nm	static quenching	diazinon	3.3 nM	2.3–100 μM	1.64	1.73%; 1.4–2.0%	Recovery %: 95.8–102.4%	hydrothermal; Te powder, NaBH_4_, CdCl_2_, Pd(OAc)_2_, TGA
Safari 2019, [74]	MPA-capped Ni-doped CdTe QDs	2 nm	MPA	ex.: 330 nm, em.: 540 nm	static quenching	pyrazinamide	0.50 µM	2–100 μM	1.70	3.13%; 2.9–3.4%	Recovery %: 97.5–101.0%	hydrothermal; Te powder, NaBH_4_, CdCl_2_, Ni(NO_3_)_2_
Wang 2019, [75]	CA-CdS QDs	8.2 nm	CA	ex.: 420 nm, em.: 570 nm	static quenching	Cu^2+^	9.2 nM	10 nM–50 µM	3.70	3.05%; 2.9–3.2%	QY: 18.82%; Recovery %: 95.80–99.70%	hydrothermal; CdCl_2_, thioacetamide, citric acid
Zhang 2019, [76]	CdTe QDs	N/A	MIPs	ex.: 350 nm, em.: 566 nm	ET	pesticide 2,4-D	90 nM	0.83–100 µM	2.08	4.92%; 4.7–5.9%	Recovery %: 94.2–107.0%	hot injection method in aqueous phase; Cd(NO_3_)_2_·4H_2_O, Te powder, NaBH_4_
Wang 2020, [24]	CdTe QDs	3.5 nm	TGA	ex.: 350 nm, em.: 560 nm	static quenching	propafenone	23 nM	0.07615–20.50 μM (0.026–7.0 μg/mL)	2.43	3.79%; 2.85–4.31%	Recovery %: 95.3–102.4%	aqueous; Te powder, NaBH_4_, CdCl_2_
Zhang 2020, [77]	S-doped Si QDs	4.77 nm	–NH_2_	ex.: 345 nm, em.: 425 nm	FRET	Fe^3+^	0.21 µM	1–20 µM	1.30	0.73%; 0.00–2.14%	QY (ref quinine): 66%; Recovery %: 90–106%	hydrothermal; APTES, trisodium citrate
Gao 2021, [78]	AuNPs-CdTe QDs@MPA	N/A	cysteamine	ex.: 365 nm, em.: 540 nm	FRET	TNT	0.24 nM	1 nM–5 μM	3.70	8.64%; 5.9–10.2%	Recovery %: 86.70–112.6%	hydrothermal; CdCl_2_, Te powder, NaBH_4_, MPA
Liu 2021, [79]	Ti3C2 MQDs (MXene QDs)	2 nm	N/A	ex.: 330 nm, em.: 430 nm	FRET	curcumin	0.20 µM	0.05–10 μM	2.30	N/A	N/A	microwave-assisted; Ti_3_AlC_2_, HF
Liu 2021, [79]	Ti3C2 MQDs (MXene QDs)	2 nm	N/A	ex.: 330 nm, em.: 430 nm	FRET	hypochlorite	5.0 µM	25–150 μM; 150–275 μM	1.04	N/A	N/A	microwave-assisted; Ti_3_AlC_2_, HF
Yang 2021, [80]	ZnCdS QDs@MIP; CdTeS QDs@SiO2	11 nm	APTES	ex.: 380 nm, em.: 530 nm	ET	ascorbic acid	0.78 µM	1–500 μM	2.70	1.23%; 1.044–1.663%	Recovery %: 96.0–99.0%	ZnCdS: aqueous refluxing method; Zn(OAc)_2_, CdCl_2_, MPA, Na_2_S; CdTeS: two-step procedure; Te powder, NaBH_4_, CdCl_2_, thiourea
Yi 2021, [81]	β-CD-MoS2 QDs	3 nm	β-CD	ex.: 295 nm, em.: 435 nm	PET	parathion-methyl	13 nM	0.01–18 mg/L (37.99 nM–68.39 μM)	3.26	3.50%; 2.67–4.42%	Recovery %: 93–105.6%	hydrothermal; Na_2_MoO_4_·2H_2_O, glutathione
Zhang 2021, [82]	CdTe QDs	5 nm	TGA	ex.: 365 nm, em.: 550 nm	static quenching	salbutamol	42 nM	62.7–209 nM	0.52	6.17%; 5.15–7.21%	Recovery %: 81.1–89.3%	hydrothermal; Te powder, NaBH_4_, CdCl_2_, TGA
Zhao 2021, [83]	NALC-CdS QDs (N-acetyl-L-cysteines)	2.03 nm	NALC	ex.: 360 nm, em.: 453 nm	ET	Cu^2+^	0.48 µM	1–25 μM	1.40	3.2%; 2.3–4.1%	QY: 34.31% (w.r.t. Rhodamine 6G in absolute ethanol); Recovery %: 99.6–101.6%	one-pot low-temperature hydrothermal route; CdCl_2_, NALC, thioaceamide
Aznar-Gadea 2022, [84]	green and red CdSe QDs	2.9 nm	N/A	ex.: 404 nm, em.: 560 nm	ET, FRET	3-nitrotoluene	10 pM	10 pM–1 mM	8.00	N/A	PL QY: 30%	hot injection method; CdO, Se solution
Aznar-Gadea 2022, [84]	green and red CdSe QDs	2.9 nm	N/A	ex.: 404 nm, em.: 560 nm	ET, FRET	4-nitrotoluene	0.50 nM	0.5 nM–1 mM	6.30	N/A	PL QY: 30%	hot injection method; CdO, Se solution
Aznar-Gadea 2022, [84]	green and red CdSe QDs	2.9 nm	N/A	ex.: 404 nm, em.: 560 nm	ET, FRET	2,3-dimethyl-2,3-dinitrobutane	0.50 nM	0.1 nM–0.1 mM	6.00	N/A	PL QY: 30%	hot injection method; CdO, Se solution
Aznar-Gadea 2022, [84]	green and red CdSe QDs	2.9 nm	N/A	ex.: 404 nm, em.: 560 nm	ET, FRET	picric acid	0.10 µM	100 nM–1 M	7.00	N/A	PL QY: 30%	hot injection method; CdO, Se solution
Wang 2022, [85]	WxOy QDs	3.35 nm	N/A	ex.: 320 nm, em.: 383 nm	IFE, FRET, PET	Tetracycline	19 nM	5–50 μM	1.00	1.82%; 0.9–3.77%	Recovery %: 97.94–109.35%	one-pot ethanol–thermal method; WS_2_, H_2_O_2_
Narasimhappa 2023, [86]	CdS QDs	N/A	N/A	ex.: 365 nm, em:. 440 nm	FRET	tetracycline	23 nM	10–100 µM	1.00	1.82%; 0.90–3.77%	QY: 55.8%; Recovery %: 80.1–106.7%	extracellular synthesis; Citrobacter freundii, cysteine, CdCl_2_
Zhong 2023, [87]	G-MoS2 QDs (glutathione)	2 nm	GSH	ex.: 360 nm, em.: 430 nm	IFE	hypochlorite	12 nM	1–30 µM	1.48	2.14%; 0.72–4.41%	QY: 6.81%; Recovery %: 99.5–102.96%	hydrothermal; Na_2_MoO_4_⋅2H_2_O, glutathione, MilliQ water
Singh 2024, [88]	Zn_3_N_2_ QDs	N/A	N/A	ex.: 320 nm, em.: 408 nm	dynamic quenching	Cu^2+^	22 nM	2.5–50 µM	1.30	2%	QY: 29.56%	hydrothermal; Zn(NO_3_)_2_×6H_2_O, NH_3_
Singh 2024, [88]	Zn_3_N_2_ QDs	N/A	N/A	ex.: 320 nm, em.: 408 nm	dynamic quenching	Mn^2+^	64 nM	0.05–5 µM	2.00	2%	QY: 29.56%	hydrothermal; Zn(NO_3_)_2_×6H_2_O, NH_3_
Velamala 2024, [89]	CsPbBr_3_@D-TA Per-QDs	13.1 nm	D-TA	ex.: 380 nm, em.: 522 nm	static quenching (aggregation)	superoxide anion	40 nM	0.125–25 µM	2.30	1.58%; 0.99–1.99%	QY: 29.8%; Recovery %: 98.43–99.81%	in-situ precipitation method; PbBr_2_, CsBr, D-TA
Zhang 2024, [90]	CdTe-MIP/SiO_2_	N/A	MIPs	ex.: 365 nm, em.: 609 nm	FRET	malachite green	3.7 nM	0.01–20 μM	3.30	1.40%; 0.5–2.5%	QY: 19.7%; Recovery %: 98.4–101.5%	hydrothermal; Te powder, NaBH_4_, CdCl_2_, MPA
Kailasa 2025, [91]	Malt@MAPbBr_3_ QDs	7.25 nm	D-maltose	ex.: 430 nm, em.: 535 nm	static quenching	γ-aminobutyric acid (GABA)	8.4 nM	0.05–10 µM	2.30	0.96%; 0.23–1.26%	QY: 23.74%; Quenching efficiency: >90%; Recovery %: 98.13–99.96%	ligand-assisted reprecipitation; MABr, PbBr_2_, 1-octadecene, oleyamine, maltose
Makwana 2025, [34]	LaSrO_3_ Per-QDs	4.45 nm	N/A	ex.: 336 nm, em.: 421 nm	FRET	bilirubin (BR)	10 nM	0.025–25 µM	3.00	0.70%; 0.48–0.86%	QY: 36.91%; Recovery %: 98.90–100.01%	microwave-assisted sol-gel method; La(NO_3_)_3_·6H_2_O, Sr(NO_3_)_2_, L-serine
Makwana 2025,[34]	LaSrO_3_ Per-QDs	4.45 nm	N/A	ex.: 336 nm, em.: 421 nm	IFE	epinephrine (EP)	17 nM	0.05–10 µM	2.30	0.48%; 0.30–0.77%	QY: 36.91%: Recovery %: 99.24–99.98%	microwave-assisted sol-gel method; La(NO_3_)_3_·6H_2_O, Sr(NO_3_)_2_, L-serine
Zhang L. 2025, [92]	MoS_2_ QDs	N/A	APBA	ex.: 320 nm, em.: 375 nm	dynamic and static quenching, IFE	glutathione (GSH)	0.48 µM	10–500 μM	1.70	3.74%; 2.45–5.52%	Assay time: 1 min; Recovery %: 98.73–103.34%	hydrothermal; Na_2_MoO_4_·2H_2_O and cysteine
Zhang L. 2025, [92]	MoS2 QDs	N/A	APBA	ex.: 320 nm, em.: 375 nm	dynamic and static quenching, IFE	ascorbic acid	0.19 µM	10–100 μM	1.00	2.71%; 0.48–6.91%	Assay time: 1 min; Recovery %: 97.44–104.7%	hydrothermal; Na_2_MoO_4_·2H_2_O and cysteine
Zhang Y. 2025, [28]	Si QDs	3 nm	N/A	ex.: 367 nm, em.: 464 nm	ET, IFE	Hg^2+^	3.0 nM	0.5–5 μM	1.00	12.27%; 4.11–18.8%	QY: 29.4%; Recovery %: 80.3–109%	microwave-assisted; trisodium citrate dehydrate, DAMO, glycerol

Abbreviations: N/A—not available; APTMS —3-aminopropyltrimethoxysilane; TOP—trioctylphosphine; CD—cyclodextrin; CX—calix [4] arene; DTCA—dithiocarbamate; FoM—figure of merit; TGA—thioglycolic acid; MPA—mercaptopropionic acid; MBA—mercaptophenylboronic acid; CCP—cationic conjugated polymers; CaDPA—calcium dipicolinate; APTES—3-aminopropyltriethoxysilane; CA—citric acid; L-AA—L-ascorbic acid; TSIM—trimethylsilylimidazole; DAMO—*N*-[3-(trimethoxysilyl)propyl]-ethylenediamine; MAA—mercaptoacetic acid; NALC—N-acetyl-L-cysteine; GSH—glutathione; rQDs—red quantum dots; gQDs—green quantum dots; QY—quantum yield; TNT—2;4;6-trinitrotoluene; DNT—2;4-dinitrotoluene; PETN—pentaerythritol tetranitrate; RDX—cyclotrimethylenetrinitramine; APBA—3-aminophenylboronic acid; D-TA—D-tartaric acid; Per-QDs—perovskite QDs; MIP—molecularly imprinted polymers; FRET—fluorescence (or Förster) resonance energy transfer; ET—electron transfer; PET—photoinduced electron transfer; IFE—inner filter effect, NPs—nanoparticles.

**Table 2:** The average LOD, log(range), and RSD were calculated from all the data shown in Table 1. (The number in brackets indicates the number of averaged data points).

Geometric mean (LOD)	38.3 nM (54)
Median (LOD)	23.0 nM (54)
Average (log(range))	2.14 (54)
Median (log(range))	1.70 (54)
Average (RSD)	3.20% (36)

The analysis of the LOD, shown in Figure 1A, has shown that proteins were detected most sensitively when the geometric mean of the LOD was equal to 8.15 nM (pLOD = 8.03). Articles detecting proteins (BR, EP, bovine hemoglobin, phycocyanin, and TRF) span the period from 2015 to 2025, with no significant change in LOD values, which indicates that there have not been many improvements made since 2015 in QD-based protein detection. However, for example, for BR detection, the sensitivity level of the system proposed by Makwana is sufficient in correlation with the normal plasma levels of this protein, which range from 3.42 to 20.52 μM [34,93]. Explosives have performed a bit weaker; however, their geometric mean was 12.9 nM, which is not much higher than the LOD for proteins. The lowest median among all the groups of analytes was calculated for environmental and industrial pollutants. This fact underlines that these three categories of analytes require highly sensitive devices to detect even trace amounts of them due to safety concerns. Small biological molecules have shown both the highest geometric mean and the highest median of the LOD, with the values being 0.360 μM and 0.275 μM, respectively. This may point out that in living organisms, these molecules are typically present at a high level, so there is no need for ultrasensitive detection. The difference between the median and the geometric mean values of the pLOD, which can be observed in Figure 1A for explosives and pollutants, suggests that there was skewness of the data, meaning there were outperformers: either positive, as with explosives, or negative, as for pollutants.

The analysis of the linear range (expressed in log scale) across the analyte categories shows a variation in sensor performance, as can be observed from Figure 1B. Explosives, proteins, and pesticides exhibit the widest working ranges, each with median log (linear range) values exceeding two orders of magnitude in width. Explosives demonstrate the broadest linear range, with an average of 3.31 and a median of 2.45. This nearly one-order-of-magnitude difference suggests the presence of outstanding sensors that skew the average upward. In contrast, drugs have the narrowest linear range, with a median of one order of magnitude (1.00), pointing out a potential limitation in the applicability of current drug sensors. This narrow range highlights the need for the further optimization of sensors designed for pharmaceutical detection. Overall, the linear range across all the analytes spans from 0.33 to 8.00 orders of magnitude [82,84], illustrating the difference in the performance of fluorescent QD-based sensors and pointing toward areas where further enhancement is required. Additionally, analyte groups, such as drugs and small biological molecules, had narrower average and median log(range) values, potentially indicating lower variability in the sensitivity of the detection methods used for these categories. On the other hand, explosives and proteins had broader log(range) values, which may reflect a wider diversity in sensor performance or detection strategies across different studies. Thus, for the system designed for the detection of explosives proposed by Aznar-Gadea and coworkers, logarithmic ranges are in the range of six (2,3-dimethyl-2,3-dinitrobutane) to eight (3-nitrotoluene) orders of magnitude, which are the widest ranges among all the analyzed papers [84].

It is also worth noting that the distinction between the geometric mean and median LOD values within each category can hint at the distribution skewness—groups with large discrepancies between these values, such as explosives, where there is a ten-fold difference, contain a few highly sensitive detection methods that significantly lower the mean.

Overall, these trends can guide future sensor development priorities, indicating areas where improved sensitivity is still needed (e.g., small biomolecules) and where reproducibility across platforms should be enhanced (e.g., explosives).

**Figure 2:**
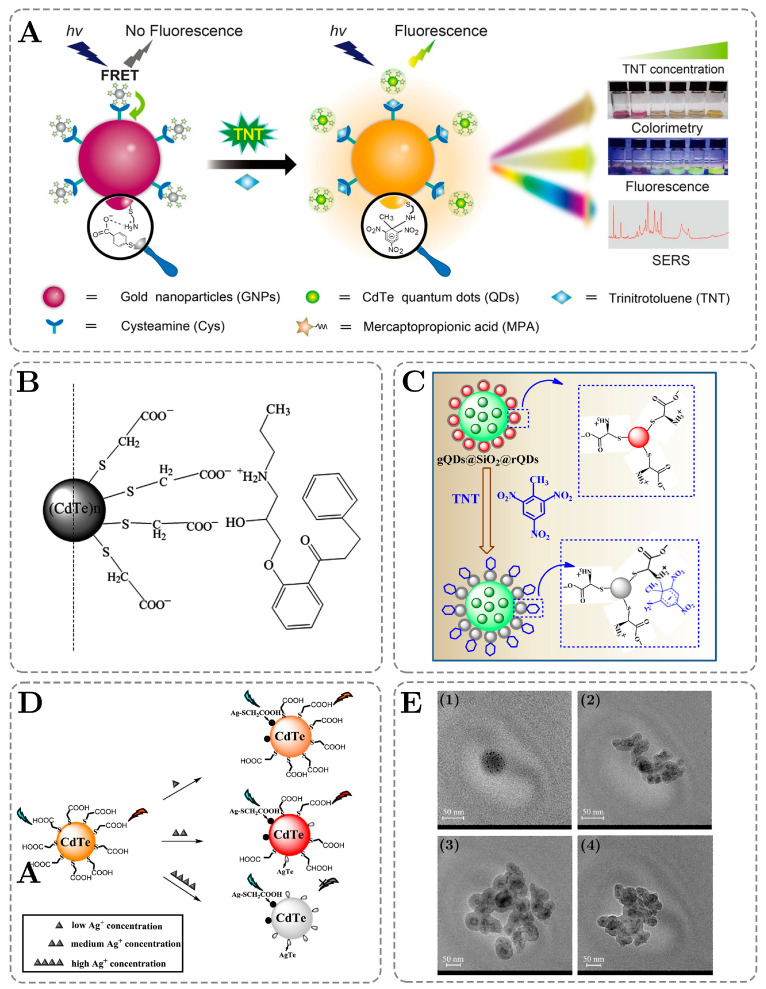
Schemes, graphs, and photographs adapted from academic papers describing QD-based fluorescence sensor mechanisms and morphology. (**A**) A graphical representation of the detection of TNT with AuNPs-CdTe QDs by fluorescence, colorimetry, and Raman spectroscopy. Adapted with permission from Gao et al. [78]. Copyright © 2021, Elsevier. B.V. (**B**) A graphical illustration of the suggested linking mode between thioglycolic acid-modified CdTe QDs and propafenone. Adapted with permission from Wang et al. [24]. Copyright © 2020, Elsevier. B.V. (**C**) A schematic representation of a TNT sensing mechanism with MPA-capped green CdTe QDs embedded into the SiO_2_ sphere and red-emitting CdTe QDs conjugated onto the SiO_2_ surface. Adapted with permission from Qian et al. [61]. Copyright © 2016, Elsevier. B.V. (**D**) A schematic representation of the working mechanism of a CdTe QD-based sensor for Ag^+^ detection in lake water. Adapted with permission from Chen et al. [23]. Copyright © 2019, Elsevier. B.V. (**E**) Transmission electron microscopy (TEM) photographs of (1) CdTe QDs, (2) CdTe QDs@SiO_2_, (3) MIP-CdTe QDs, and (4) non-imprinted particles (NIP)-CdTe QDs designed for tetrabromobisphenol-A detection. Adapted with permission from Feng et al. [71].

Gao and coworkers have developed a sensor for TNT detection and have tested three detection methods, which include colorimetric detection, fluorescence, and Raman spectroscopic detection. In the sensor design process, they utilized cysteamine-modified AuNPs with a 532 nm absorption wavelength and MPA-capped CdTe quantum dots with a 540 nm emission wavelength. As shown in Figure 2A, at first, AuNPs-QD assemblies are formed due to electrostatic interactions between QDs@MPA and AuNPs@Cys. The Fluorescence of QDs is quenched via the FRET mechanism. After TNT is added, the QDs-AuNPs composite is disassembled, which turns on fluorescence and colorimetric signals. An increase in the fluorescence intensity due to an increase in the TNT concentration can be observed with the naked eye, as shown in Figure 2A. Apart from that, the strong affinity of TNT to Cys leads to the formation of the Meisenheimer complex and the subsequent self-aggregation of AuNPs, which generates a strong Raman enhancement and allows for the sensitive detection of TNT in complex matrices. The LOD for the fluorescence detection of TNT achieved by Li and Ren’s groups was 0.24 nM, and the linear range of the sensor spans 3.70 orders of magnitude, from 1 to 5 µM [78].

Wang and coworkers have developed a CdTe QD-based system for the detection of propafenone, with an LOD of 23.2 nM and a linear range of 76.15 to 20.5 μM. Figure 2B demonstrates the suggested binding model between propafenone and CdTe QDs in acidic media, where a connection happens due to electrostatic attraction and hydrogen bonds, which results in the formation of larger ionic association complexes. This leads to the quenching of the fluorescence of QDs by hindering the electron transfer in the semiconductor nanocrystal from an excited to a ground state.

Qian and coworkers have developed a TNT sensor based on MPA-capped CdTe gQDs and Lcys-capped CdTe rQDs whose mechanism of operation is shown in Figure 2C. Due to the electron deficiency of TNT and the electron richness of Lcys, Meisenheimer complexes were formed between TNT and Lcys ligands through the acid–base pairing interactions, hydrogen bonding, and electrostatic co-interactions. The quenching of the fluorescence emission of rQDs at 625 nm is induced by the electron transfer from rQDs to the aromatic ring of TNT, while the emission of interior gQDs at 508 nm stayed almost unaffected. The variation in the intensity ratio of the dual emissions led to a significant shift in the fluorescence color, enabling the visual detection of TNT on-site. The sensor has exhibited an LOD of 3.3 nM and a wide linear range of 10 to 8 μM [61].

Chen and coworkers have designed a sensor for the selective and sensitive detection of Ag^+^ ions in lake water. The device is based on TGA-capped CdTe QDs, which were synthesized via a one-pot aqueous method. Green, orange, and red QDs were obtained from potassium tellurite, cadmium acetate, and thioglycolic acid by changing the reaction time and temperature. Experimental results have shown that green-emitting QDs demonstrate the highest sensitivity, with their LOD being 5 nM, while red-emitting QDs exhibit the widest linear detection range, from 5 to 200 µM. As can be seen from Figure 2D, the interaction between Ag^+^ and QDs is based on the formation of an Ag–S bond with TGA, and the sensor has different responses for different silver ion concentrations. At a low Ag^+^ concentration, there was no significant change in the emission wavelength of QDs; however, the fluorescence intensity was slightly weakened. Medium Ag^+^ concentrations have led to a red shift in the emission wavelength of orange QDs, while at a high Ag^+^ content, the fluorescence of QDs was completely quenched [23].

Feng and coworkers have developed a sensor for tetrabromobisphenol-A detection in e-wastes based on MIP-coated CdTe QDs. Figure 2E demonstrates the morphologies of bare QDs (1), QDs@SiO2 (2), MIP-QDs (3), and NIP)-QDs (4) through the TEM images. The increase of the MIP-coated particle diameter compared to the bare QDs can be observed from Figure 2E(1,2) to Figure 2E(3), demonstrating that the MIP-QDs possessed a big surface area with well-defined imprinted sites for binding template molecules. From the images, it can be also seen that the particles were spherical and were dispersed uniformly. Figure 2E(3,4) illustrate that the MIP-QDs and the NIP-QDs exhibited similar morphologies, with their shape and size becoming slightly rough and irregular due to sol-gel polymerization throughout the synthesis process. The designed sensor has shown a LOD of 0.552 nM (0.3 ng/mL) and a linear range of 1.0 to 60.0 ng/mL [71].

**Figure 3:**
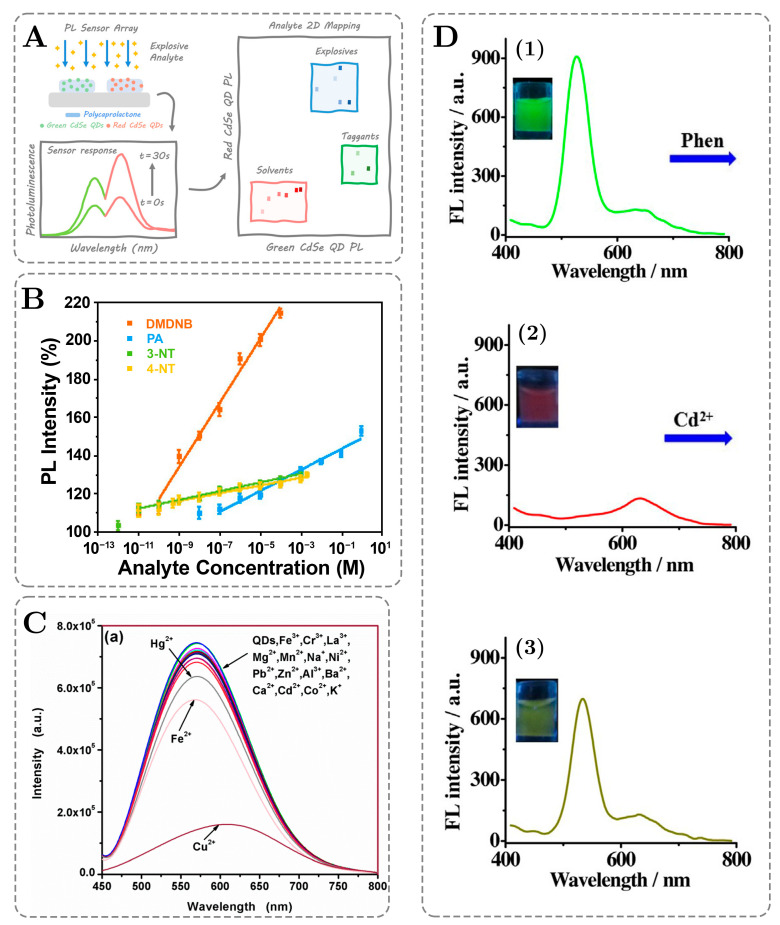
Examples of efficient fluorescence-based sensing with the application of QDs: (**A**) A schematic mechanism of a CdSe QD-based sensor for the detection of four explosives (3-nitrotoluene (3-NT), 4-nitrotoluene (4-NT), 2,3-dimethyl-2,3-dinitrobutane (DMDNB), and picric acid (PA)). (**B**) Calibration plots for the detection of four explosives (3-NT, 4-NT, DMDNB, and PA). Adopted under Creative Common CC BY license from Aznar-Gadea et al. [84]. Copyright © 2022, American Chemical Society. (**C**) Fluorescence spectra illustrating the impact of different metal ions on CA-CdTe QD fluorescence during the Cu^2+^ sensing. Adopted under Creative Common CC BY license from Wang et al. [75]. Copyright © 2019, MDPI, Basel, Switzerland. (**D**) Photographs and fluorescence spectra of (1) rQDs@SiO_2_@gQDs, (2) rQDs@SiO_2_@gQDs-Phen (20 μM), and (3) rQDs@SiO_2_@gQDs-Phen-Cd^2+^ (2 μM) produced by the nanosensor for Cd^2+^ detection. The excitation wavelength was 365 nm. Adapted with permission from Qian et al. [64]. Copyright © 2017, Elsevier. B.V.

Some other representative examples of fluorescence-based sensing with QDs are shown in Figure 3 below.

The best-performing sensor was developed by Aznar-Gadea and coworkers, and its mechanism of operation is schematically shown in Figure 3A. At first, red- and green-emitting CdSe QDs were embedded into polycaprolactone as a host polymer matrix. Authors have determined that the signals of green and red QDs towards the same compounds are different, which allows for the two-dimensional (2D) mapping of experimental results where the intensity of red QDs is plotted against the intensity of green QDs, as depicted in Figure 3A. Such 2D maps allow for building fingerprints for each compound, which leads to a decreased number of false positives and increases the assay performance towards multiple types of explosives. Figure 3B demonstrates the calibration curves of four tested analytes, namely 3-NT, 4-NT, DMDNB, and PA. All the compounds, except picric acid, can be detected at a subnanomolar concentration, with the lowest LOD achieved for 3-NT at 10 pM. From the calibration curves, it can also be concluded that linear ranges for the different types of analytes span from six to eight orders of magnitude, with the widest range being achieved for 3-NT [84].

Wang and coworkers proposed a sensor for the selective detection of Cu^2+^ ions by the citric acid-capped CdS QDs. The authors managed to achieve a high performance, with the LOD of the sensor being 9.2 nM and the linear range spanning 3.70 orders of magnitude, from 10 nM to 50 µM. Figure 3C illustrates the selectivity test for Cu^2+^ ions with 17 different heavy transition metals, from which it can be observed that only copper ions quench the fluorescence of the QDs, while other metals do not have a significant effect on the fluorescent signal. Also, the red shift of the fluorescence signal towards Cu^2+^ can be noticed. The red shift suggests that there is a formation of a new energy level below the conduction band of the CdS, which can be attributed to the photochemical reduction of adsorbed copper ions by CA-CdS QDs. From an XPS analysis, which is not shown here, the authors have proved the presence of both Cu^2+^ and Cu^+^ and concluded that the quenching of fluorescence takes place due to the deposition of CuS on the surface of the QDs [75].

Qian and coworkers have developed a Cd^2+^ nanosensor based on CdTe red- and green-emitting QDs (rQDs and gQDs) in the form of rQDs@SiO2@ to detect cadmium ions at such low concentrations as 0.17 nM. In their study, 1,10-Phenanthroline (phen) was used as the sensing molecule. Figure 3D illustrates the fluorescence spectra of the as-obtained rQDs@SiO2@gQDs (Figure 3D(1)), after the addition of phen (Figure 3D(2)), and after the addition of Cd^2+^ ions (Figure 3D(3)). Upon excitation with a 365 nm wavelength, the as-obtained rQDs@SiO2@gQDs showed two highly resolved emission peaks at 525 and 635 nm, while the fluorescence intensity of the gQDs loaded on the surface of the hybrid spheres was significantly quenched. The addition of the Cd^2+^ ions to the system resulted in a noticeable recovery of the fluorescence because of the removal of phen ligands from the surface of gQDs due to the strong chelating of phen with Cd^2+^. Thus, it is possible to use cadmium cations as the fluorescence activation switch, disrupting the photo-induced hole transfer process and restoring the fluorescence of the gQDs. Apart from changes in the spectra, there is a color change following each step described above, as can be observed from Figure 3D. Since the intensity of the fluorescence of the rQDs stayed unaffected all the time, it can be used as a reference signal, thus providing an in-system correction for environmental effects. Meanwhile, the fluorescence of the gQDs changed, and the quenching efficiency in the pH range of 6 to 9 took the values from 94.33 to 97.79% [64].

## 3. Chemiluminescence of QDs: Applications in Sensing and Detection

Chemiluminescence in nanomaterials is a process where light is emitted from the nanostructure, like in the QDs in this review article, during the chemical reaction, rather than from the remission of the absorbed light. It has raised the interest of scientists as a promising tool for sensing and detection techniques. The classification of the types of chemiluminescence varies, but mostly, the following three types are classified: general chemiluminescence (CL), bioluminescence (BL), and the most common in sensing, electrochemiluminescence (ECL).

General chemiluminescence involves the emission of light due to chemical reactions that are not related to biological or electrochemiluminescent triggers. For instance, in forensic science, the oxidation of luminol can be used for the detection of blood. Typically involving enzyme-catalyzed reactions, bioluminescence is found in living organisms, such as fireflies, glowworms, and some marine animals. Finally, electrochemiluminescence is triggered by an electrochemical reaction, where the periodical applied potential triggers the light emission. It is commonly used in analytical chemistry for sensitive analyses, like immunoassays [94].

While fluorescence was and remains the most common technique for sensing purposes, during the 2000s, researchers demonstrated the capabilities of chemiluminescence for detection. For instance, in 2007, Heyou Han’s research group developed a method that allowed the determination of hydrogen peroxide with an LOD of 60 nM by electrochemiluminescence from thiol-capped Cd-Te QDs. This method was novel in terms of its analytical performance compared to previous reports for the analyte. Another well-recognized paper published a few years later by Lin et al. focused on the nitrite sensing by CL of fluorescent carbon dots induced with peroxynitrous acid, which acquired a detection limit of 53 nM. Together, these and other papers demonstrated the potential of CL quantum dots in analytical chemistry [95,96].

There are also some review papers published in the last several years that investigate the chemiluminescence of QDs, mostly from qualitative sight. The newest one by Yang et al. mainly explores the diverse roles of dots in electrochemiluminescent bioanalysis, while also presenting some quantitative comparisons. In contrast, the 2021 review by Tzani et al. investigates the mechanisms and applications of direct and indirect chemiluminescence. It highlights the recent improvements in the enhancement of CL efficiency with various chemical structures. The last one, a review paper by the Yi Lv group outlines the recent advancements of liquid-phase CL systems, where quantum dots are primarily used as a final emitting species via direct redox reactions of chemiluminescent resonance energy transfer, emphasizing their direct analytical applications aside from roles as catalysts or enhancers [94,97,98].

Recent advancements in this field involve the application of new materials, such as perovskite and MXene quantum dots, for the development of highly sensitive platforms for biosensing, as well as the exploration of multiplex detection using multicolor techniques. Table 3 provides detailed information regarding the chemiluminescence–QD-based sensors developed for sensing purposes. Specific nanostructures, brief preparation methods, analyte molecules, and important analytical parameters, such as the LOD, QDs’ size, emission wavelength, and linear parameters, are all covered to help identify the trends and observe the common elements of sensing [99,100,101].

The information gathered in Table 3 spans the past 13 years of quantitative analytical research. The largest class of analytes found in the reviewed papers consists of clinical biomarkers, while other analytes include small molecules, toxins, and contaminants, as well as pesticides and herbicides. Some of the abbreviations used are **Ab** for antibody, **black-P** for black phosphorus, **FL** and **PL** for the wavelengths reported for fluorescence or photoluminescence exclusively, **IgG** for immunoglobulin G, **LR** for leucine–arginine, **MIL** for materials of institute Lavoisier (adapted from French), **N/D** for values that are not present explicitly in the articles, and **RET** for resonance energy transfer.

**Table 3:** Analytical performance of QD–chemiluminescence-based sensors.

Year, Last Name	Nanostructure	Preparation (RS: Reflux Synthesis. TGA: Thioglycolic Acid. 3-MPA: 3-Mercaptopropionic Acid)	Analyte	LOD	Range	Size (nm)	Emission λ (nm)	RSD	R^2^	Recovery
Han 2007, [95]	thiol-capped CdTe QDs	RS: CdCl_2_, TGA, NaHTe	hydrogen peroxide	60 nM	(2.0 × 10^−7^–1.0 × 10^−5^) M	3.32	620	4.80%	0.999	N/D
Wang 2012, [102]	SiO_2_-QD-Ab2	Solution aging: CdCl_2_, methiopropamine, NaHTe	human IgG (HIgG)	0.58 fM	(1.0 × 10^−10^–1.0 × 10^−5^) g/L	N/D	705	4.60%	0.996	83.8–120.6%
Fang 2012, [22]	CdSe QDs	RS: CdCl_2_, TGA, NaHSe	α-fetoprotein	70 fM	(5.0 × 10^−9^–1.0 × 10^−4^) g/L	2.5	575 (PL)	1.50%	N/D	N/D
Liu 2014, [103]	CdSe QDs	RS; CdCl_2_, HMP, MPA, Na_2_SeO_3_, N_2_H_4_	dopamine	3.0 nM	(3.0 × 10^−9^–1.0 × 10^−5^) M	N/D	541	4.30%	N/D	104.5–106.9%
Dong 2014, [104]	CdSe@ZnS QDs	RS: CdCl_2_, TGA, NaHTe	thrombin	1.4 fM	(1.0 × 10^−14^–1.0 × 10^−10^) M	4.5	550	2.3% to 4.1%	0.995	100.8–102.4%
Zhang 2015, [105]	CdS QDs	RS: CdCl_2_, methiopropamine, thioacetamide	microcystin-LR	2.8 pM	(1.0 × 10^−8^–5.0 × 10^−5^) g/L	3.5	646 (PL)	3.36%	0.999	97.7–101%
Wang 2015, [106]	CdTe/CdS coresmall/shellthick QDs	RS: CdCl_2_, methiopropamine, NaHTe	Cu^2+^	20 nM	(1.0 × 10^−7^–1.0 × 10^−5^) M	4.8	710	3.3%	0.998	93.3–104.6%
Dong 2017, [107]	CdTe QDs (QDs)-embedded mesoporous silica nanospheres	N/D	carcinoembryonic antigen	1.7 fM	(1.0 × 10^−9^–8.0 × 10^−5^) g/L	3.45	676	5.93%	0.996	96.24–105.26%
Zhao 2017, [108]	MoS_2_-QDs	RS; MoS_2_, Pd-Au CHs	lipopolysaccharide	0.07 fg/mL	(1.0 × 10^−13^–5.0 × 10^−5^) g/L	4.2	625	1.53%	0.998	N/D
Wu 2017, [109]	Ag_2_S:Mn QDs	RS; 3-MPA, acetic acid, AgNO_3_, Na_2_S, Mn(Ac)_2_	laminin	3.6 fM	(1.0 × 10^−8^–1.0 × 10^−4^) g/L	4	626	N/D	0.997	96.08–105.56%
Dong 2017, [110]	Si QDs	RS: aminopropyl trimethoxysilane, trisodium citrate	target DNA	16 aM	(1.0 × 10^−16^–1.0 × 10^−12^) M	6.72	475	3.50%	0.994	N/D
Yang 2018, [111]	CdTe QDs	RS; CdCl_2_, trisodium citrate dehydrate, Na_2_TeO_3_, MPA, NaBH_4_	cardiac troponin-I antigen	19 aM	(1.1 × 10^−12^–1.1 × 10^−5^) g/L	5	650	0.92%	0.999	93.6–109.7%
Hu 2018, [112]	CdSe QDs (CdSe QDs)	RS: sodium sulfite, selenium powder, CdCl_2_, 3-MPA, N_2_H_4_	hydrogen peroxide	0.50 µM	(5.0 × 10^−7^–5.0 × 10^−4^) M	8	470, 610	4.90%	0.972	87.6–104.3%
Zhang 2018, [113]	Black-P QDs	Sonication: black phosphorus powder, N-Methyl-2-pyrrolidone	dopamine	22 pM	(1.0 × 10^−10^–5.0 × 10^−8^) M	8.2	610	1.30%	0.994	103 to 105%
Wang 2018, [114]	CdTe QDs	RS: NaHTe, CdCl_2_, TGA	clopyralid	4.1 pM	(2.0 × 10^−11^–3.5 × 10^−8^) M	8	N/D	1.78–2.42%	0.997	93.3–105.7%
Lei 2018, [115]	SnS_2_ QDs	Hydrothermal method: SnCl_4_, L-cysteine	anti-CMV pp65	0.33 fM	(1.0 × 10^−15^–1.0 × 10^−7^) M	5	665	N/D	0.996	97.78–108.20%
Gao 2019, [116]	CdTe QDs	RS; CdCl_2_, Na_2_TeO_3_, 3-MPA, NaBH_4_	ochratoxin A	0.42 pM	(5.0 × 10^−10^–5.0 × 10^−5^) g/L	3	585	2.90%	0.999	96.1–100.7%
Liu 2019, [117]	Black-P QDs	Sonication: black phosphorus powder, N-Methyl-2-pyrrolidone	lysozyme	2.0 fM	(1.0 × 10^−10^–1.0 × 10^−7^) g/L	8.2	N/D	0.56%	0.991	98–102%
Du 2020, [118]	MOF-5-wrapped CdS quantum dot	RS: CdCl_2_, Na_2_S, mercaptopropionic acid	cardiac Troponin I	0.21 fM	(1.0 × 10^−11^–1.0 × 10^−6^) g/L	5.4	685475	9.25%	0.993	98.0–104.7%
Chen 2020, [119]	CdTe QDs@NH_2_-MIL-88(Fe)	RS: CdCl_2_, Na_2_TeO_3_, trisodium citrate, NaBH_4_, 3-MPA	malathion	0.91 fM	(1.0 × 10^−12^–1.0 × 10^−6^) g/L	7.5	689 (1.8 eV)	7.00%	0.993	99.2–110%
Xu 2020, [120]	Multiwall, carbon-nanotube-enhanced, molecularly imprinted QDs	N/D	cyfluthrin	0.11 nM	(2.0 × 10^−7^–1.0 × 10^−3^) g/L	N/D	N/D	2.70%	0.998	86.0–98.6%
Liu 2021, [121]	CdS QDs	RS: 3-MPA, CdCl_2_, NaOH, Na_2_S, ethanol.	17β-estradiol	0.11 pM	(1.0 × 10^−11^–1.0 × 10^−8^) g/L	N/D	N/D	1.1–2.5%	0.99	98.9–118.4%
Feng 2021, [122]	MIL-53(Al)@CdS QDs	N/D	trichlorfon	5.1 pM	(1.0 × 10^−11^–1.0 × 10^−4^) M	<10	517	2.50%	0.997	97–105%
Rasoulzadeh 2021, [123]	AgInS_2_ QDs	RS; AgNO_3_, InCl_3_, sodium citrate, TGA, thiourea, deionized water, ethanol.	glutathione	0.28 nM	(1.0 × 10^−9^–5.0 × 10^−6^) M	2	680	3.10%	0.994	98–101%
Zhao 2021, [124]	Black-P-CdTe QDs	RS; CdCl_2_, BP-nanosheets, Na_2_TeO_3_, 3-MPA, NaBH_4_	miRNA-126	29 aM	N/D	N/D	709	1.19%, 2.85%	0.998	98.8–104%
Yang 2021, [99]	Ir NRs@CdS QDs	RS: CdCl_2_, Na_2_S, L-cysteine	ethyl paraoxon	1.7 pM	(5.0 × 10^−12^–5.0 × 10^−8^) M	N/D	543	2.38%, 2.68%	0.995	91.9–108.0%
Jia 2022, [125]	CdSe@CdS QDs	N/D	ochratoxin A	2.2 nM	(1.0 × 10^−6^–1.0 × 10^−4^) g/L	15	634	2.1–6.5%	0.994	97.3–105.6%
Li 2022, [101]	CH_3_NH_3_PbBr_3_ QDs@SiO_2_	Ligand-assisted reprecipitation method; PbBr_2_, CH_3_NH_3_Br, APTES, toluene	aflatoxin B1	27 fM	(1.0 × 10^−11^–1.0 × 10^−5^) g/L	N/D	566	1.2-2.8%	0.997	101.7–106.7%
Liu 2022, [126]	luminol/MoS_2_ QDs@zeolitic imidazolate framework-8	RS; MoS_2_, luminol, ZnNO_3_ solution	miRNA21	15 aM	N/D	2	N/D	N/D	0.998	N/D
Yang 2023, [127]	ECL-RET sensor with TGA-capped CdS QDs	RS: CdCl_2_, TGA, Na_2_S	EGFR T790M ctDNA	3.4 aM	(1.0 × 10^−17^–1.0 × 10^−13^) M	5.7	497 (FL)	2.6%	0.991	N/D
Yang 2023, [127]	ECL-RET sensor with TGA-capped CdS QDs	RS: CdCl_2_, TGA, Na_2_S	EGFR T790M ctDNA	8.1 aM	(5.0 × 10^−17^–1.0 × 10^−13^) M	5.7	497 (FL)	2.6%	0.994	N/D
Li 2023, [100]	Mo_2_TiC_2_ QDs	Acid etching, alkaline treatment, and microwave-assisted synthesis; Mo_2_TiAlC_2_ powder, NH_4_HF_2_, TMAOH, ultrapure water.	miRNA-27a-3p	1.0 fM	(1.0 × 10^−15^–1.0 × 10^−8^) M	2.7	594	2.00–2.59%	0.992	89.1–104.2%
Liu 2024, [128]	CdSe@ZnS/MXene@NaAsc	N/D	uric acid	18 pM	(1.0 × 10^−10^–1.0 × 10^−4^) M	4	N/D	2.81%	0.987	88.40–94.65%
Gong 2025, [129]	AgInZnS QDs	RS: methiopropamine, AgNO_3_, In(Ac)_3_, Zn(Ac)_2_	carcinoembryonic antigen	16 fM	(5.0 × 10^−14^–1.0 × 10^−8^) M	N/D	620	1.13%	0.994	95.5–103.3%

The geometric average of the detection limits is 1.1 × 10^−13^ M or 109 fM, which is noticeably lower compared to 26 and 38 nM for phosphorescence and fluorescence, respectively, which share approximately the same geometric mean value. This is attributed to the absence of excitation light in CL: since chemiluminescence is produced via chemical reaction, background interference, and autofluorescence are significantly minimized, leading to a greater signal-to-noise ratio. Moreover, half of the detections had an LOD better than 27.2 fM, with the lowest included in this review being 3.4 aM, for the sensing of cancer mutations on a linear DNA analyte with the application of CdS dots in 2023 by Yang et al. [127].

The arithmetic average for the width of the linear range, calculated as log_10_(*c*_max_/*c*_min_), results in 4.27 orders of magnitude, with the best-reviewed being 8.7 orders of magnitude for the detection of lipopolysaccharide by Zhao et al. [108], with MoS_2_ dots in 2017. There is some positive correlation between the pLOD and the width of the linearity, meaning that for a lower LOD, there is an expected wider linear range; however, the correlation is not so strong, with a Pearson coefficient of +0.58.

The central values for the emission wavelength and the size of the quantum dots are 615 nm and 4.8 nm, respectively. Compared to fluorescence and phosphorescence, 615 nm for chemiluminescence is almost no different than phosphorescence, but around one standard deviation higher than the same value for fluorescence. The size of the particles is also greater for CL than for the other two luminescence types, but its variance is too big for a conclusion based on the given sample size.

Regarding the reproducibility of the methods, the mean value of the relative standard deviation for the measurements and the linearity of the calibration curve is equal to 5.1% and 0.9945, respectively. For the recoveries, the absolute average deviation from 100%, in percent recovery, is 5.05%.

It is noted that almost all sensors, with only a few exceptions, have utilized electrically induced CL with electrodes, rather than direct chemiluminescence from spontaneous reactions like luminol and hydrogen peroxide. This selection is based on ECL’s capacity to precisely regulate the timing and localization of light generation, which enhances the precision of the measurements. It also deserves to be mentioned that ECL offers a wider dynamic range and higher sensitivity while preserving the simplicity and stability of traditional CL sensors due to less dependence on experimental conditions for reactions. Moreover, ECL-QDs can be used not only as emitter material but also as coreactants or ECL-RET components, which enables them to be used in new sensing mechanisms unavailable for conventional methods [97,130]

Aside from the analytical applications of the nanomaterials described, we could not find articles that utilize chemiluminescent quantum dots for clinical applications.

**Figure 4:**
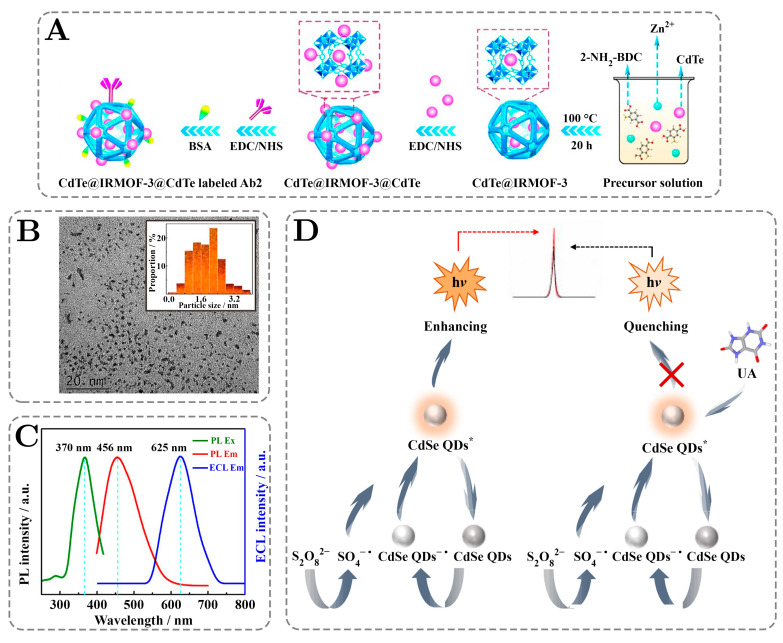
Analytical papers based on luminescence sensing techniques. (**A**) The synthesis of an ECL immunosensor with dramatically enhanced CdTe-QD intensity based on isoreticular metal–organic framework-3 (IRMOF-3) with a narrow RSD and a low LOD. Adapted with permission from Yang et al. [111]. Copyright © 2018 American Chemical Society. (**B**) A TEM image with a size distribution insert of MoS_2_ QDs used in a biosensor for the atto-molar detection of microRNA with a 12.7-fold increase in ECL efficiency. Adapted with permission from Liu et al. [126]. Copyright © 2022 American Chemical Society. (**C**) Normalized PL excitation, PL emission, and ECL spectra of MoS_2_ quantum dots applied in the eco-friendly, sensitive detection of lipopolysaccharide, achieving the widest linear range, of 8.7 orders of magnitude, among all the reviewed articles. Adapted with permission from Zhao et al. [108]. Copyright © 2017, American Chemical Society. (**D**) A possible, and common for ECL, mechanism of the molecularly imprinted electrochemiluminescence sensor used for the sensing of uric acid with a ratiometric analysis. Adapted with permission from Liu et al. [128], © 2023 Elsevier B.V. All rights reserved.

**Figure 5:**
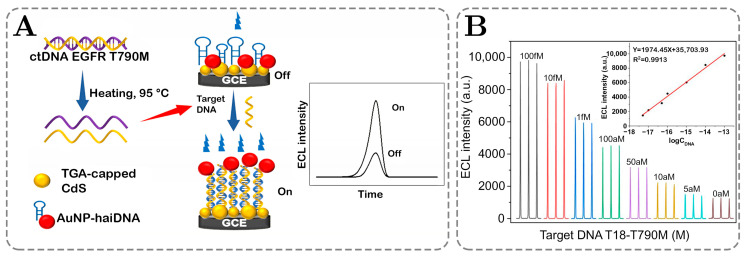
(**A**,**B**) ECL-resonance energy transfer system allowing detection of cancer mutations on linear DNA analytes with atto-molar sensitivity. Adopted with permission from Yang et al. [127]. Copyright © 2023 American Chemical Society.

The articles featuring chemiluminescent sensors with outstanding analytical performances and intuitive visual representations are compiled in Figure 4 and Figure 5 below to summarize key trends and regularities in chemiluminescent sensing using quantum dots.

Figure 4A presents a synthetic pathway for the fabrication of a highly sensitive electrochemiluminescent biosensor based on CdTe QDs enriched with a metal–organic framework to enhance the detection of a key biomarker for myocardial infarction–cardiac troponin-I (cTnI). The novelty of the sensor lies in the signal amplification method, where an isoreticular metal–organic framework-3 is integrated with CdTe dots to utilize it not only as a high-capacity carrier but also as a coreactant accelerator, which is crucial for ECL. The synthesis involved the usage of 2-amino terephthalic acid as the organic ligand to encapsulate CdTe within IRMOF-3, which significantly increased the packing of QDs and improved the emission intensity by making possible the conversion of persulfate into highly reactive sulfate radicals. Then, using this composite as a labeling probe in a sandwich immunoassay, an ultra-low LOD of 19.2 aM and a wide linear range of seven orders of magnitude were acquired. The mechanism applied not only enhanced the quantum dots’ ECL performance but also ensured superior stability and selectivity, which is required for broader applications [111].

Figure 4B provides a transmission electron microscopy image of the molybdenum disulfide quantum dots that were used in the fabrication of the luminol/MoS_2_ QDs@Zeolitic imidazolate framework-8 for the sensitive detection of microRNA-21. A high level of this RNA is a marker of cancer or cardiac disease. As seen on the inset, the distribution of particle sizes is not uniform and is clustered in a wide range between 0.8 nm and 2.8 nm. While variability in the dots’ diameter is not considered to be a desirable phenomenon as it may cause batch-to-batch variability and inhomogeneous ECL signal, for the given case, the detection was very sensitive, with a limit of 14.6 aM. Typically, luminol is used in a pair with hydrogen peroxide to produce the chemiluminescent signal; however, as was shown in this paper, the application of MoS_2_ dots as a coreactant to luminol enhances the efficiency of the signal up to 12.7 times [126].

Continuing with MoS_2_ dots, Figure 4C demonstrates the spectra for this type of QDs in terms of their excitation by photoluminescence and emission by both photoluminescence and chemiluminescence. The PL excitation peak is the most blue-shifted compared to any emission peak; however, ECL is more red-shifted than the PL emission peak. Under electrochemical excitation, charges can localize on or near QD surface states more readily than under optical excitation. Emissions from these lower-energy states or excitons partially “trapped” at the surface tend to appear red-shifted relative to the band-edge emission that dominates photoluminescence. In their paper, Zhao et al. demonstrated the synthesis of non-toxic MoS_2_ dots that are used in sensing lipopolysaccharide, which is considered a contaminant in biotechnology and research. The achieved linearity range of Pd-Au, convex, hexoctahedron-immobilized QDs is 8.7 orders of magnitude, which is the largest found for this review value. The acquired limit of detection is 0.07 fg/mL [108,131].

Figure 4D demonstrates the mechanism of the common ECL from the work of Liu et al., who have synthesized an MXene@NaAsc-enhanced sensor based on CdSe@ZnS for the detection of uric acid, with an achieved LOD of 18.13 pM. This method, like many others, utilizes coreactants with QDs to achieve an enhanced ECL signal. This is possible due to the generation of reactive intermediates and energy transfer facilitation caused by coreactants. Hydrogen peroxide, persulfates, oxalates, and tripropylamine are typical examples of coreactants. In the given case, persulfate ion is used. Under applied potential CdSe@ZnS QDs and persulfate ions, both gain an electron from a reduced radical species:

CdSe@ZnS QDs + e^−^ = CdSe@ZnS QDs^−^•

S_2_O_8_ + e^−^ = SO_4_^−^• + SO_4_^2−^

The reduced QDs then react with a sulfate radical anion to initiate an excited state in the QDs (denoted as QDs*), which then relaxes back to the ground state with the emission of a photon:

CdSe@ZnS QDs^−^•+ SO_4_^−^• = CdSe@ZnS QDs* + SO_4_^2−^

CdSe@ZnS QDs* = CdSe@ZnS QDs + h**ν**

However, in the presence of uric acid, a portion of the quantum dot radicals is oxidized, preventing the formation of their excited state; consequently, a reduction of ECL signal is observed:

CdSe@ZnS QDs^−^• + Uric Acid = CdSe@ZnS QDs + Uric Acid^−^•

The concentration of the analyte is then found by the linear fitting of the ratio of the ECL signal to the applied current and the decimal logarithm of uric acid concentration [128].

Figure 5A,B present an electrochemiluminescent biosensor based on cadmium sulfide quantum dots, which have applications in detecting cancer-related mutations in circulating tumor DNA (ctDNA). The sensor is designed to identify the epidermal growth factor receptor (EGFR) T790M mutation, a critical marker in non-small-cell lung carcinoma. Typical methods, like polymerase chain reaction (PCR), require a significant number of extra nucleotides on both sides of the region of interest for successful detection, whereas the developed sensor can work with short segments (18–100 nucleotides) of DNA with terminal mutations. It consists of a glass carbon electrode with immobilized thioglycolic acid-CdS QDs and AuNP-labeled hairpin DNA (AuNP-haiDNA).

As shown in Figure 5A, during the “off state” of the sensor, Au nanoparticles cause the quenching of ECL emission from QDs due to high proximity; however, when the target DNA is introduced into the system, it selectively binds to complementary hairpins on the nanoparticles, which forces structural changes, decreasing the proximity of AuNP-haiDNA to the glassy carbon electrode (GCE), causing an increase in the emission signal. The performance of the sensor depends on the mutation position: when the terminal position is analyzed, an LOD of 3.4 aM with a linear range of 50 aM to 100 fM is achieved; in comparison, a higher detection limit of 8.1 aM but a wider linear range of 10 aM to 100 fM is achieved for the middle mutation position. Since complementary DNA is used in this method, it shows excellent selectivity. As a result, the method could contribute to highly sensitive, rapid, and cost-effective ctDNA mutation detection for cancer diagnostics.

Figure 5B depicts a common setup for chemiluminescent measurements and calibration. As seen, the vertical axis represents the ECL emission intensity in arbitrary units for varied concentrations of the analyte, whereas the horizontal axis indicates the time. The inset graph provides the semilogarithmic calibration plot of the emission intensity versus the log of the concentration of a standard solution. The short-term emission is induced by forcing an electrochemiluminescent reaction with an applied potential, which is then recorded. To reduce the variability of the signal, the signals from a few stimulations are averaged. The same steps are taken on each of the standard solutions. Finally, the average maximum ECL emission intensity is plotted against the concentration on the semilogarithmic plot to achieve a linear relationship for calibration [127].

## 4. Phosphorescence of QDs: Applications in Sensing and Detection

Because of their unique optical properties and long-lived excited states, phosphorescent quantum dots (PQDs) became one of the prominent molecular sensing tools. Another reason might be the obstacles that are caused by fluorescent quantum dots and yet are solved by PQDs, such as delayed fluorescence, since it has the ability to undergo intersystem crossing from singlet to triplet states [132].

In addition to that, fluorescent sensor arrays have several disadvantages, such as autofluorescence and scattering light originating from endogenous biomolecules. PQDs solve these issues, particularly Mn-doped ZnS QDs, by emitting orange phosphorescence (~590 nm) via energy transfers from the ZnS host lattice to the Mn^2+^ dopants, followed by a spin-forbidden transition from the excited triplet state (^4^T_1_) to the ground state (^6^A_1_), which achieves a longer emission lifetime. A longer emission lifetime allows us to effectively filter out short-lived background fluorescence and Rayleigh scattering. Furthermore, the difference between the excitation and emission wavelengths is significant because of Mn-doped ZnS QDs’ large Stokes shifts, which in turn reduce the spectral overlap and enhance the signal clarity. These differences can be seen in Table 4: the excitation occurs at 295–304 nm, while the emission stays around 590 nm. Because of all of these advantages, PQDs demonstrate great results in detecting hyaluronic acid, melamine, heparin, and other biomolecules [132,133,134,135].

For a long time, fluorescent quantum dots dominated the molecular sensing field. However, He et al. showed the benefits of PQDs for the detection of enoxacin in 2008 by demonstrating a 58.6 nM detection limit [136]. In the following years, researchers started discovering the potential of PQDs in molecular sensing during metal ion detection and environmental pollutant detection [137,138,139]. Most PQDs are ZnS-based because of their wide band gap and surface passivation ability. Moreover, the protection of the quantum dot core by ZnS from the environment plays a significant role in the reduction of photobleaching and an increased emission time, leading to better sensing abilities [140].

In addition to ZnS properties, the diameter of PQDs also should be considered since it affects various properties, such as the emission wavelength, functionalization surface area, and photostability. Therefore, we analyzed various PQDs in Table 4, and the most common radius is 3.5 nm, which completely agrees with the theoretical calculations, according to which, the smaller the QDs’ size, the more intense the emissions for the detections of analytes with low concentrations.

According to 54 studies on PQDs, we found the following analytical conclusions. The geometric average LOD is 26 nM, with a median of 38 nM. The average PQD diameter is 4.6 nm (median 3.7 nm). The values for the average and the median RSD are 3.9% and 3.4%, respectively. This data shows how efficient and reproducible PQDs are. In addition, R^2^ (0.996 for average and median) demonstrates the consistency of PQDs.

Another important thing to consider is analyzing the best-performing PQDs, along with the worst ones, to understand their strengths and weaknesses. Therefore, we took PQDs corresponding to these parameters and examined their synthesis/functioning mechanisms.

**Figure 6:**
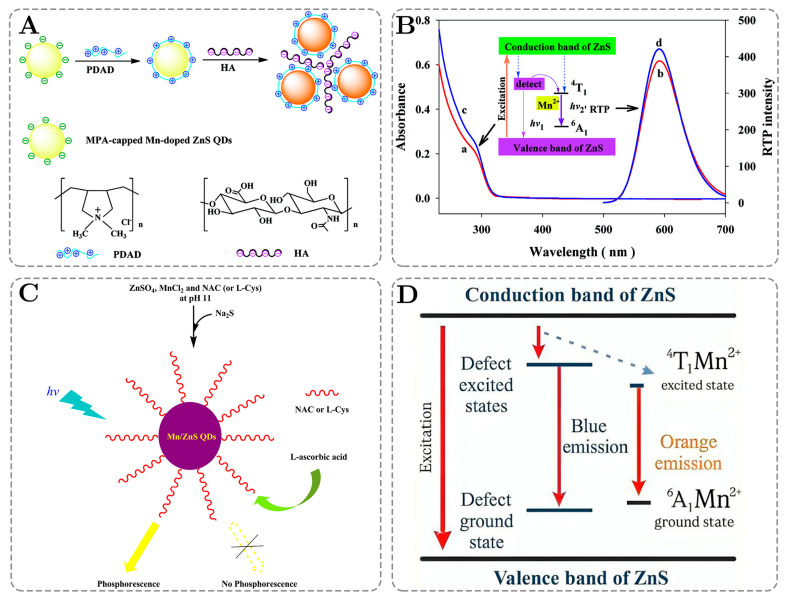
(**A**) Schematic illustration of fabrication of PDAD–Mn–ZnS QDs for HA detection. Adopted under Creative Common CC BY license from Li et al. [134]. Copyright © 2018. (**B**) UV/vis spectra (curves a and c) and RTP emission spectra (curves b and d) of Mn–ZnS QDs (10 mg mL^−1^; curves a and b) and PDAD–Mn–ZnS QDs (10 mg mL^−1^; curves c and d). Adopted under Creative Common CC BY license from Li et al. [134]. Copyright © 2018. (**C**) Schematic illustration of sensor design. Adapted with permission from Bian et al. [147]. Copyright © 2013, Elsevier. B.V. (**D**) Schematic of emission mechanism of TG-Mn-ZnS QDs.

The best-performing PQDs, demonstrating the lowest LOD (79 fM), utilized Mn-ZnS QDs capped with mercaptopropionic acid (MPA). To positively charge QD nanohybrids, these PQDs were functionalized with poly (diallyldimethylammonium chloride) (PDAD), a cationic polymer, as illustrated in Figure 6A. The purpose of the positive charging was to improve their room-temperature phosphorescence and minimize background interference by utilizing strong electrostatic interactions after the addition of the analyte, HA, since it is negatively charged [134]. In Figure 6B, we analyzed the optical properties of these PQDs. According to the figure, these PQDs have a maximum absorption peak at 295 nm (curve a) and an emission peak at 590 nm (curve b), where the transition of electrons from the triplet excited state ^4^T_1_ to the ^6^A_1_ ground state of Mn^2+^ is responsible for the emission. It is also important to note that PDAD does not change the absorption/emission properties of Mn-ZnS QDs since PDAD-functionalized Mn–ZnS QDs have the same absorption/emission peak at 295/590 nm as unmodified Mn–ZnS QD [134].

**Table 4:** Analytical performance of sensing based on phosphorescence quantum dots (PQDs).

Year, Family Name	Nanostructure	Preparation	Analyte	LOD	Range	Diameter (nm)	RSD (%)	R^2^	Excitation Wavelength (nm)	Emission Wavelength (nm)	Binding Molecule
Wu 2010, [132]	Mn-doped ZnS QDs	CS	Glucose	3.0 µM	10 µM–1 mM	3	3.2	0.9855	290	595	1-ethyl-3-(3-dimethylaminopropy)carbodiimide (EDC)/N-hydroxysuccinimide (NHS)
Wang 2010, [141]	Mn-doped ZnS QDs	CS	ascorbic acid	9.0 nM	0.05–0.8 µM	4.2	4.8	0.9913	337	595	Sodium Tripolyphosphate
Wang 2011, [135]	ZnS QDs	Combination of colloidal and template-assisted synthesis	2,4,6-trinitrotoluene	50 nM	0.05–1.8 µM	5	3.5	0.9902	316	580	3-Aminopropyltriethoxysilane-functionalized manganese
Yu 2011, [142]	Mn-doped ZnS QDs	CS	DNA	0.10 µM	0.08–12 mg L^−1^	3.5	3.7	N/D	316	590	methyl violet
Zhang 2013, [143]	Mn-doped ZnS QDs	N/D	DNA	27 pM	0 to 45 nM	N/D	3.73	0.9991	N/D	581	N/D
Wu 2013, [138]	Mn-doped ZnS QDs	Combination of colloidal and template-assisted synthesis	trypsin	40 nM	0.1–1.2 μM	3.8	N/D	0.9917	280	590	bovine serum albumin (BSA)
Dan 2013, [144]	Mn-doped ZnS QDs	N/D	domoic acid (DA)	67 nM	0.25−3.5 μM	N/D	0.65	0.99	300	590	Polyethyleneimine
Tan 2013, [145]	Mn-doped ZnS QDs	CS	bovine hemoglobin	38 nM	1.0 × 10^−7^–5.0 × 10^−6^ mol L^−1^	7	1.8	0.998	340	585	3-Mercaptopropyltriethoxysilane (MPTS)
Wang 2013, [146]	Mn-doped ZnS QDs	CS	catechol	53 nM	0.5–5 μM	N/D	3.2	0.9962	316	595	Sodium Tripolyphosphate
Bian 2013, [147]	Mn-doped ZnS QDs	CS	L-ascorbic acid	0.72 µM	2.5–37.5 µM	9	1.4	0.9983	315	583	N-acetyl-L-cysteine
				14 µM	2.5–47.5 μM	9	1.8	0.995	306	580	L-cysteine
Bian 2014, [148]	Mn-doped ZnS QDs	CS	Co^2+^	60 nM	1.25 × 10^−6^–3.25 × 10^−5^ M	10	2.3	0.9989	315	583	N-acetyl-L-cysteine
Zhu 2014, [149]	Mn-doped ZnS QDs	Microwave-assisted synthesis	indapamide	0.89 µM	1.5–80 µM	4.2	3.4	0.995	310	585	MPA
Gong 2014, [150]	Mn-doped ZnS QDs	CS	clenbuterol	12 nM	5–1000 ng·mL^−1^	3.5	2.9	0.9985	295	590	MPA
Zhang 2015, [151]	Mn-doped ZnS QDs	CS	protamine	33 nM	0.2–3.0 µg mL^−1^	3.5	2.76		295	590	MPA
Bi 2015, [152]	Mn-doped ZnS QDs	CS	DNA	0.14 µM	0.2–20 mg L^−1^	2.5	1.7	0.998	295	590	MPA
Gong 2015, [153]	Mn-doped ZnS QDs	CS	phosphopeptides	N/D	1.6–2800 ng mL^−1^	3.5	2.5	0.9969	295	590	MPA
Zhang 2015, [154]	Mn-doped ZnS QDs	CS	quercetin	0.16 µM	0.1–6.0 mg mL^−1^	3.5	4.6	0.996	295	590	MPA
Ertas 2015, [155]	Mn-doped ZnS QDs	CS	double stranded DNA/ idarubicin	0.48 µM	2.0–20.0 μM	3.5	4.35	0.9943	290	590	L-cysteine
Bian 2015, [156]	ZnS QDs	CS	histidine	0.74 µM	1.25–30 µM	10	0.65	0.997	315	589	Co^2+^-adsorbed N-acetylL-cysteine (NAC)
Zhang 2015, [157]	Mn-doped ZnS QDs	CS	glucose	7.0 µM	20–800 µM	2.7	0.5	0.996	310	597	N/D
Chang 2016, [133]	Mn-doped ZnS QDs	CS	human serum albumin	10 nM	0.02–1.0 µM	N/D	N/D	0.9799	301	602	Rhodamine B (Rh B)
Chen 2016, [158]	Mn-doped ZnS QDs	CS	Pb^2+^ ions	2.2 nM	1-100 μg L^−1^	3	0.54	0.9962	280	590	glutathione
Gong 2016, [159]	Mn-doped ZnS QDs	CS	DNA	46 pM	15 μg L^−1^–40 mg L^−1^	3	1.9	0.998	295	590	MPA
Liu 2017, [160]	Mn-doped ZnS QDs	CS	trypsin	1.8 nM	0.88–15.6 µg mL^−1^	N/D	5	0.993	316	598	Cytochrome c (Cyt c)
Lv 2017, [161]	Mn-doped ZnS QDs	CS	miRNA-21	1.6 nM	8–80 nM	3.5	3.4	0.989	295	590	ROX-DNA
Zhang 2017, [162]	Mn-doped ZnS QDs	N/D	alkaline phosphatase	N/D	0.001–0.1 U/L	N/D	N/D	0.999	312	600	PNPP (p-nitrophenylphosphate)
Zhang 2017, [163]	Mn-doped ZnS QDs	Hydrothermal/CS	thiram	25 nM	50 nM–2.5 μM	5	N/D	0.9975	312	590	mercaptosuccinic acid (MSA)
Pacheco 2017, [164]	Mn-doped ZnS QDs	CS	warfarin	4.7 µM	1.07 × 10^−5^ M–4.50 × 10^−5^ M	2.7	N/D	0.997	276	584	l-cysteine (L-cys)
Zhang 2017, [165]	Mn-doped ZnS QDs	CS	patulin	0.32 µM	0.43-6.50 µmol L^−1^	10.25	4.2	0.9945	324	585	3-Mercaptopropyltriethoxysilane (MPTS)
Deng 2017, [166]	Mn-doped ZnS QDs	CS	permanganate anions (MnO_4_^−^)	0.24 µM	0.5–100 μM	7.75	2.95	0.9981	315	585	L-cysteine
Lv 2017, [167]	Mn-doped ZnS QDs	CS	transgenic 35S promoter DNA	4.0 nM	12–300 nM	5	7.9	0.997	295	590	DNA
Li 2018, [134]	Mn-doped ZnS QDs	CS	alkaline phosphatase		0.15–18 U L^−1^	4	4.2	0.994	295	590	Eu^3+^
Li 2018, [168]	Mn-doped ZnS QDs	CS	Micrococcal nuclease base		2 × 10−3–8.0 × 10−2 U mL^−1^	3.5	5.2	0.993	295	595	DNA-ROX
Wei 2018, [169]	Mn-doped ZnS QDs	CS	2,4,6-trichlorophenol	35 nM	0.1–30 μmol L^−1^	N/D	4	0.99	320	594	magnetite (Fe_3_O_4_)
Liu 2018, [170]	Mn-doped ZnS QDs	CS	adriamycin	0.45 µM	0.5–64.0 µM	4.5	N/D	0.9932	316	585	poly(diallyldimethylammonium chloride) (PDDA)
Li 2018, [134]	Mn-doped ZnS QDs	CS	hyaluronic acid (HA)	79 fM	0.08–2.8 μg mL^−1^	4	2.1	0.995	295	590	MPA
Zou 2018, [171]	Mn-doped ZnS QDs	CS	copper(II)	6.0 nM	0.01–12 μM	3.6	3.2	0.994	316	590	alginate
Qin 2018, [172]	Mn-doped ZnS QDs	CS	resveratrol	10 nM	0.03–14 µM	3.5	2.7	N/D	295	595	MPA
Luo 2019, [173]	Mn-doped ZnS QDs	CS	4-nitrophenol	60 nM	0.1–100 μM	4	4.6	0.998	295	590	N/D
Chen 2019, [174]	Mn-doped ZnS QDs	CS	cephalexin	2.3 nM	2.5–50 μg·L^−1^		5	0.9985	295	590	thioglycolic acid (TGA)
Zhao 2019, [175]	Mn-doped ZnS QDs	CS	picric acid	6.1 nM	2.0–180 ng mL^−1^	5.8	2.2	0.9985	316	600	Melamine (MA)
Miao 2019, [137]	Mn-doped ZnS QDs	CS	melamine	1.6 µM	0.005–6 mM	4	2.6	0.997	297	590	ssDNA
Liu 2020, [176]	Mn-doped ZnS QDs	CS	tetracyclines	8.6 nM	50–1.5 × 10^5^ nM	3.8	6	0.995	289	583	L-Cysteine
Jayasinghe 2020, [177]	Mn-doped ZnS QDs	CS	aflatoxins	11 pM	2–20 µg L^−1^	2.3	20	0.9947	290	594	Polyethylene glycol (PEG)
Jinadasa 2020, [178]	Mn-doped ZnS QDs	CS	As(III), As(V)	0.12 nM	0–20 μg L^−1^	N/D	10	N/D	289	595	(3-aminopropyl) triethoxysilane and an As(III) ionic
Lv 2020, [179]	Mn-doped ZnS QDs	CS	phenol	2.1 µM	5.0 to 55 μmol L^−1^	5.5	3.7	0.9984	330	600	3-mercaptopropyltriethoxysilane (MPTS)
Liu 2021, [180]	Mn-doped ZnS QDs	CS	thyroxine	2.0 nM	4.85 nmol/L–1.59 μmol/L	3.5	N/D	0.999	295	590	MPA
			carbamazepine	3.4 nM	7.9 nmol/L–1.555 μmol/L	3.5	N/D	0.995	295	590	
Qin 2021, [181]	Mn-doped ZnS QDs	CS	alkaline phosphatase		0.2−10 U/L	3.65	N/D	0.993	310	680	pyrophosphate
Chen 2021, [182]	Mn-doped ZnS QDs	CS with sol-gel synthesis	norfloxacin	2.5 nM	1–90 μg L^−1^	5.5	7	0.9993	300	590	magnetite (Fe_3_O_4_)
Fan 2021, [139]	Mn-doped ZnS QDs	CS	chlorpyrifos	0.89 µM	0–80 μM	N/D	1	0.99	275	600	N/D
Kong 2023, [183]	Mn–ZnS QDs@g-C_3_N_4_	N/D	2,4,6-trinitrotoluene	0.56 µM	0–12 μM	3.6	6	0.997	318	582	mercaptoethylamine (MEA)
Yang 2025, [184]	Mn-doped ZnS QDs	CS	lead (II)	2.6 nM	5 × 10^−6^–100 mM	2.12		0.9979	360	596	1-thioglycerol
Summary of Average/Median Values in Table 4											
Median LOD (M)	3.8 × 10^−8^	Median Diameter (nm)	3.7	Median RSD (%)	3.4	Median R^2^	0.996	Median Excitation wavelength (nm)	300	Median Emission wavelength (nm)	590
Geometric Average LOD (M)	2.6 × 10^−8^	Average Diameter (nm)	4.6	Average RSD (%)	3.9	Average R^2^	0.996	Average Excitation wavelength (nm)	304	Average Emission wavelength (nm)	590

The abbreviations used are CS for colloidal synthesis, DNA for deoxyribonucleic acid, MPA for 3-mercaptopropionic acid, ROX for 6-carboxy-x-rhodamine, and N/D for the values that are not explicitly mentioned in the papers.

The PQDs with the worst performance (the LOD is 13.8 μM) were synthesized using the wet-chemical approach, where ZnSO_4_ * 7H_2_O, MnCl_2_ * 4H_2_O, and N-Acetyl-L-cysteine (NAC) were mixed in a 50 mL reaction volume. According to the article, the optimal pH for PQD synthesis was 11, which was regulated by 1.0 M NaOH. Using nitrogen bubbling, dissolved oxygen was removed so that the Na_2_S * 9H_2_O solution could be injected into the mixture to start the synthesis of NAC-Mn/ZnS QDs. After purification, centrifugation, and vacuum drying, the desired PQDs were obtained. This synthesis mechanism is briefly described in Figure 6C. However, in that case, they replaced NAC with cysteine as the stabilizing agent. It seems like the most important factor for the LOD in this case is the substitution of NAC by cysteine since the LOD significantly increases from 0.72 to 13.8 μM [147].

The PQDs with the smallest diameter (2.12 nm) demonstrate absorption at 300–350 nm and emission at 596 nm, according to Figure 6D. Because of the strong coupling between the 3d^5^ electrons of Mn^2+^ and the s-p electrons of the ZnS host, the electron transition occurs from ^4^T_1_ to ^6^A_1_ of the Mn^2+^ ions doped within the ZnS lattice, which makes the emission 100% pure dopant [184].

It can be concluded that the PQDs with the lowest LOD utilized electrostatic interaction as one of their techniques to achieve very high sensitivity by using PDAD, while the performance of the PQDs with the highest (worst) LOD significantly suffered from the substitution of the binding molecule joining NAC to cysteine. Therefore, it can be clearly seen how modifications and ligand choices play a significant role in the detection performance of PQDs.

## 5. Quantitative Analysis of Relationships Between Sensitivity and Other QD Parameters in QD-Based Sensing

**Figure 7:**
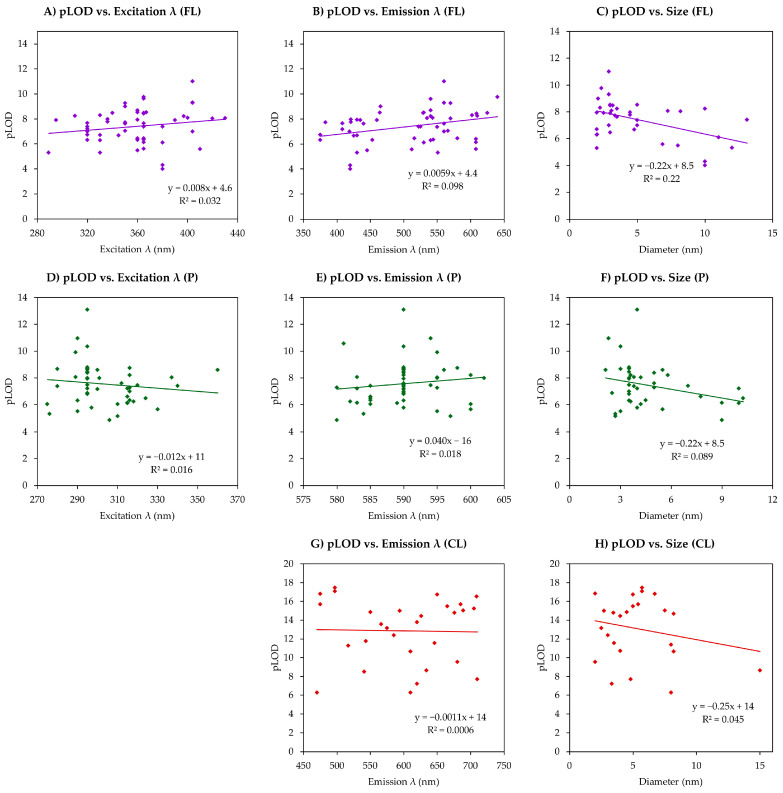
Scatter plots presenting the relationships between the negative decimal logarithm of the detection limit and the emission wavelength, excitation wavelength, and quantum dots’ size for the three types of QDs considered for analysis in this review paper. Parts (**A**–**C**) display fluorescent QDs (FL), parts (**D**–**F**) phosphorescent (P), and parts (**G**,**H**) chemiluminescent (CL).

In order to understand which QD parameters are likely to impact/improve the QDs’ sensitivity (LOD) in analysis, we have represented the relationships between the pLOD (M) and the major parameters of quantum dots reported in the literature, including excitation wavelengths, emission wavelengths, and QDs’ average diameter or size. We graphically represent those relationships in Figure 7. We have also calculated the linearity coefficients displayed on the plots, as well as the Pearson correlation coefficients displayed in Table 5.

As can be concluded from the data, the wavelength of excitation light has little influence on the pLOD (hereinafter, detection limit) for both fluorescence- and phosphorescence-based quantum dots. However, there is a significant difference between the typical values for the excitation wavelengths for these two methods. For fluorescence, the values are generally gathered around 359 ± 31 nm, with many values exceeding 350 nm. In contrast, the phosphorescent excitation wavelengths are typically equal to 304 ± 17 nm, with almost no data points above 350 nm, which is noticeably shorter than those of FL.

As a matter of observation, the phosphorescent systems require higher energy excitation wavelengths compared to fluorescent ones by almost 50 nm, which seemingly populates triplet excited states and defect states more efficiently, since P is based on them. However, we were unable to find sources that explicitly explain why P-based QDs have lower excitation wavelengths than FL-QDs. The variation of values is narrower for phosphorescent systems, with distinct clusters around 295 nm and 315 nm that are attributed to well-known classes of phosphorescent nanomaterials, like Mn^2+^-doped ZnS quantum dots. It is also worth noting that the best detection limits for P-based sensors were achieved at higher energy excitation light.

There is a small but noticeable positive correlation between sensitivity and the emission wavelength both in fluorescence and phosphorescence, though to a smaller extent in the latter one, with Pearson correlation coefficients of +0.31 and +0.13 for FL and P, respectively. This may be related to a reduced autofluorescence from biological samples at lower energy emission light levels. Additionally, QDs that emit red light tend to be bigger and, therefore, brighter. For the fluorescence, longer emission wavelengths also reduce the interference from the excitation light, which can be close in wavelength. In phosphorescence, a longer lifetime of the excited triplet state helps the separation of the signal from short-lived noise, which is particularly useful for the detection of trace analytes. The mean value for the emission wavelength resulted in 509 ± 74 nm for fluorescence-based sensing and 590 ± 5 nm for phosphorescent-based sensing. Similar to the emission light, the excitation light’s wavelengths are more clustered for phosphorescence, with distinct groups around 304 ± 17 nm for Mn-doped ZnS dots due to less available nanomaterials with tunable phosphorescence. Chemiluminescent QDs show no meaningful correlation with r = −0.02 and data points that are scattered across the plot. For chemiluminescence, sensitivity is more dependent on the efficiency of the chemical or the electrochemical reaction, rather than on the emission wavelength. The average emission wavelength for CL is 602 ± 75 nm.

As expected for quantum dots as nanomaterials with tunable properties, the strongest correlation is observed between the detection limit and quantum dots’ size. In each of the three types of luminescence, there is a negative correlation, showing that higher sensitivity is achieved with smaller QDs. The correlation is the strongest for FL, with r = −0.47, weaker for P, with r = −0.30, and minimal for CL, with r = −0.21. For fluorescent quantum dots, a smaller size leads to a higher surface-to-volume ratio, making QDs more responsive towards surface interactions, such as analyte binding, pH levels, or ion concentrations. Moreover, smaller dots have stronger confinement, which increases the energy gap and promotes radiative energy relaxations, enhancing their sensitivity to the environment. This size correlation is not so firm for phosphorescence and chemiluminescence as these systems are more dependent on triplet state dynamics or reaction kinetics than on particle size. The average QD sizes obtained are 4.9 ± 3.2 nm for FL, 4.6 ± 2.2 nm for P, and 5.3 ± 2.8 nm for CL.

Table 6 below presents the arithmetic mean and median values for the pLOD, emission wavelength, excitation wavelength, and particle diameter.

As seen, the pLOD values for fluorescence (FL) and phosphorescence (P) are nearly identical, with averages of 7.4 and 7.6 and medians of 7.6 and 7.4, respectively. This indicates that FL has slightly more high-sensitivity results than low-sensitivity ones, while in the case of P, the average is elevated by a few exceptionally low LOD values reported in the literature. As mentioned above, the comparable sensitivity of phosphorescent QDs, despite their lower emission intensity, is explained by their delayed emission, which allows for the elimination of the main drawbacks of fluorescence—namely, autofluorescence, Rayleigh scattering, and stray light—through techniques such as time-resolved phosphorescence [133,185]. The same explanation goes to chemiluminescent nanomaterials, which do not have any excitation light at all, which allows measurements with reduced background noise.

The average, 304 nm, and median, 299 nm, values for the excitation wavelength for phosphorescence are lower compared to these of fluorescence, which are 359 and 360 nm, respectively, whereas the emission value is greater. The emission wavelengths for chemiluminescence, which are 602 nm on average and 615 for the median are pretty much comparable to those of phosphorescence, since both methods experience lower energy electron transitions, though for slightly different reasons: triplet transition for P, and a lower energy intake for CL. As a matter of observation, the size of chemiluminescent quantum dots is slightly larger than that of fluorescent or phosphorescent quantum dots. This is related to the fact that larger CL-QDs possess better performance, making them more desirable in research. With the increased particle size, the degree of matching between the energy produced during the chemical reaction and the energy gap of the QDs is maximized, which means that the production of excited state QDs might be more efficient. Furthermore, since the quantum yield of the luminescence in QDs is inversely proportional to the confinement energy, and the confinement energy decreases with the increasing size, it must be the case that luminescence efficiency should correspondingly increase [186]. Other papers also verify that for larger QDs, the emission intensity of the ECL is greater [187].

## 6. Perspectives of Improvements in Sensing

Quantum dots exhibit excellent luminescent properties; however, due to surface trap sites, they are prone to the deterioration of their properties, such as the quantum yield and stability, which is still considered a challenge for researchers [188]. For perovskite QDs, limited stability hinders their characterization and slows down their technological progress [189,190]. Some of the common methods for QD stabilizations, as described by Sanjayan et al., include ligand exchange/doping, coating silica shells, and developing polymer encapsulations [191].

Yu et al. have utilized a selenium coating to stabilize colloidal HgTe QDs by tuning precursor reactivity. Se stabilization allowed for enhanced colloidal stability, surface passivation, and a variety of possibilities for further doping. As a result, the researchers have constructed adjusted *p-i-n* HgTe colloidal QD infrared photodetectors that exhibit an ultra-low dark current of 3.26 × 10^−6^ A cm^−2^ at −0.4 V and a room-temperature-specific detectivity of 5.17 × 10^11^ Jones at wavelength ≈ 2 µm, which leads to almost one order of magnitude improvement [192].

A group working with Mattoussi has developed a strategy to stabilize CsPbBr3 perovskite QDs (Per-QDs) by using coatings of polyzwitterion polymers, which were prepared via a nucleophilic addition reaction between amine-modified sulfobetaine anchors and solubilizing motifs of poly(isobutylene-*alt*-maleic anhydride). By their nature, Per-QDs tend to degenerate upon storage, processing, or testing, as well as lose their colloidal properties, absorption, and emission features after exposure to such polar media as alcohols or water. Typically used capping molecules include oleamine and oleic acid, which bind weakly to the Per-QDs and desorb quickly. That also contributes to the instability of Per-QDs. Authors list several methods of Per-QD stabilization, which include using additional ligand species, like alkyl-zwitterion, dimethylammonium, or octylphosphoric acid; ligand exchange with such small molecules as dimethylammonium bromide or 2,2′-iminodibenzoic acid; and embedding Per-QDs with a protective macroscale coating, including a polystyrene matrix. The hydrophilicity of Per-QDs was promoted by PEG blocks or zwitterion motifs, and a variety of prepared multifunction polymers allowed for a range of colloidal QDs, plasmonic nanomaterials, and magnetic NPs that demonstrate excellent photophysical properties and outstanding stability [189].

Apart from the stabilization of QDs, coating and/or ligand doping also enhances their biocompatibility [188,193]. Some of the reports of the stabilization of semiconductor QDs to improve their biocompatibility include works presented by Wang et al. and Nie et al.; however, both have utilized toxic synthetic components and media such as TOPO (trioctylphosphine oxide) or HDA (hexadecylamine) [194,195]. In contrast, Parani et al. have synthesized gelatin-stabilized, semiconductor (CdSe/CdS/ZnS)-core/double-shell quantum dots. In their study, they used the described QDs as fluorescent probes for *in vitro* HeLa cell imaging, and the experimental results showed that gelatin stabilization decreased the cytotoxicity by 50%. Moreover, gelatin stabilization and the CdS/ZnS double shell increased the photoluminescence quantum yield from around 20% (just CdSe QDs) to almost 60% (CdTe/CdS/ZnS/gelatin). The properties of the prepared QDs were preserved for a year [193].

The stabilization and reduction of cytotoxicity for any biosensing materials remain a hot topic in research, especially in QD-based sensors as they tend to use toxic components (such as transitional metals) that are released upon the degradation of the dots. However, there are reported attempts to improve the situation. For instance, in their study, Ali et al. demonstrate that the encapsulation of MPA-CdTe QDs with polyethylene glycol (PEGylation) shifts the half maximal inhibitory concentration (IC_50_) from less than 0.12 nM to more than 0.21 nM after 24 h, leading to the possibility of applying almost twice the concentration of QDs. Furthermore, the cell viability was detected to be 65% for the PEGylated sensor compared to 45% for the bare MPA-CdTe at the same 0.2 nM concentration. Although the specific sensing results are not mentioned, they reported the acquisition of a brighter luminescence signal from the nanomaterial, and, therefore, a lower LOD, while still preserving the viability of a cell. Such improvement is attributed to the ability of poly(ethylene furanoate (PEF) to shield surface-bound Cd^2+^ release and to neutralize the surface charge, almost doubling the tolerable concentration [196]. Another interesting observation was presented by the research group of K. Kim, who conducted a cytotoxicity assessment of CdSe/ZnS QDs. The diameter of the yellow dots’ core was 5.2 nm, while for green QDs it was 2.2 nm. They have demonstrated that bigger, yellow QDs, despite having dimmer light emission per particle compared to green ones, can remain non-toxic for the cells at concentrations up to 179 μg/mL, whereas green dots are lethal for the cells at 28 μg/mL. However, the usage of yellow dots decreased the signal-to-noise ratio by 33%, which means that in practical sensing, scientists must balance the choice: brighter (green) QDs might deliver a better LOD at the cost of a narrow cytotoxicity window [197]. Finally, for cytotoxicity, the study by Chen et al. focused on the cellular intake, intracellular dynamics, and short-term toxicity of PEG-coated quantum dots. They found that branched 6-armed PEG-amine/mPEG QDs have almost 10 times lower cellular uptake compared to linear PEG-QDs, which remarkably reduces the short-term cytotoxic effects; however, they still have long-term delayed effects related to the accumulation of radical oxygen species in their intracellular space, leaving the potential space for future modifications to resolve that issue [198]. Hence, while there is still a lack of direct quantitative comparison studies between raw and modified QDs, it can be seen that decreased cytotoxicity can lead to sensing improvements by increasing the maximum allowed biological sensing concentration of luminophore, leading to a brighter luminescence response. Future works might focus on combining these approaches to further limit the release of the toxic degradation products of QDs, which is also directly linked to the stability of a nanomaterial, as discussed above.

## 7. Application of Machine Learning in QD-Based Luminescent Sensors

The implementation of machine learning (ML) techniques into QD-based sensing platforms allows for achieving the enhancement of sensor performance, data interpretation, and automation. ML and deep learning-based sensing systems have a promising potential for biomarker detection [199,200]. For example, a research group of Qi and He have developed a QD- and magnetic bead (MB)-based ultrasensitive device for the detection of protein biomarkers, which was tested for interleukin-6 (IL-6). The authors have developed a novel deep learning model, ATTBeadNet, based on the UNet3+ architecture, which was used to count the magnetic beads on the images with a higher accuracy than previous AI models, such as ImageJ (code can be found at: https://github.com/foodszhang/ATTBeadNet; accessed on 9 July 2025). ATTBeadNet enables the accurate counting of fluorescent and total magnetic beads by processing paired fluorescence (FL) and “reference field” (RF) images. This bead counting is crucial for determining protein biomarkers, such as IL-6, since one Ab1-bound, streptavidin-coated capture MB binds one IL-6. The model identifies beads involved in a newly formed sandwich immunocomplex, which consists of antibody-functionalized MBs, IL-6, and horse-radish peroxidase (HRP)-labeled secondary antibodies. CdS QDs are utilized as signal reporters due to their narrow spectral width and high optical stability, which allow for signal clarity enhancement and strongly benefit FL microscopy. The use of RF images instead of traditional bright-field (BF) images improves signal-to-background ratios, which enables sensitive and automated detection in the assay. The designed system allows for the ultrasensitive quantification of IL-6 with an outstanding LOD of 3.1 fM and a linear range of 5 to 100 fM [199].

In another research article, Saren’s group applied an optimized machine learning approach, the OPCA model. It enhances principal component analysis (PCA) by incorporating a neighborhood rough set algorithm to improve gene feature selection for gastrointestinal tumor classification. This model has yielded high classification precision (over 99%) and accuracy (above 94%) for both colon and gastric cancer datasets. The selected gene features were then applied to design a multicolor CdSe/ZnS QD-based immunobiosensor for the simultaneous detection of four tumor markers, namely α-fetoprotein (AFP), cancer embryonic antigen (CEA), cancer antigen 19-9 (CA19-9), and carbohydrate antigen 125 (CA125). Each marker was conjugated to a different color-emitting CdSe/ZnS QD, which allowed for multiplexed detection with a minimized spectral overlap. The OPCA model has led to a more efficient feature extraction, which improved the performance of the biosensor in tumor classification. The sensor was successfully tested, and the working ranges for all four biomarkers are from 2.0 to 51.5 ng/mL. Such integration of ML with QD-based biosensing demonstrates a promising direction for the high-throughput, accurate detection of cancer biomarkers and the clinical diagnosis of gastrointestinal tumors [200].

A research group of Liu and Wang have applied ML to a CsPbBr_3_ perovskite QD (Per-QD)-based device for the rapid and accurate detection and sterilization of food pathogens, such as *E. coli*, *S. aureus*, *S. typhimurium*, *Listeria monocytogenes* (*L. monocytogenes*), and *P. aeruginosa*. The ML algorithm, namely a Support Vector Machine (SVM), was utilized for the analysis of relative signal intensity changes. The concentration ranges at which pathogens can be detected by the proposed system span four orders of magnitude from 1 × 10^3^ to 1 × 10^7^ CFU/mL. The LODs were also reasonably low for each of the five bacteria: *E. coli*—94 CFU/mL, *S. aureus*—117 CFU/mL, *S. Typhimurium*—93 CFU/mL, *L. monocytogenes*—136 CFU/mL, and *P. aeruginosa*—100 CFU/mL. The interaction of the QDs with different bacteria suspensions and various concentrations caused different degrees of fluorescence quenching to occur due to the aggregation of the Per-QDs, which in turn led to changes in color. After this, relative signal intensity changes, ΔRGB (red-green-blue), were captured by the Color Grab application on a smartphone and analyzed with SVM, which allowed for the precise identification and sensitive quantification of five bacterium types. Studies have also revealed that the designed systems possess strong bactericidal properties, with the deactivation efficiency of *E. coli* and *S. aureus* being more than 99% at 30 min after detection [201].

## 8. Conclusions

Sensing and bio-sensing methods based on QD applications demonstrated high (typically nanomolar or lower LOD molarity) sensitivity in the detection of a variety of analytes. The luminescence of QDs measured in sensing applications can employ fluorescence, phosphorescence, and chemiluminescence. QD-based methods using chemiluminescence demonstrated, on average, the highest sensitivity, typically in the sub-picomole range of molarity, which is five or six orders of magnitude higher with regard to sensitivity or lower with regard to the LOD in comparison with the average sensitivity/LOD of the QD-based sensing methods, where fluorescence or phosphorescence are measured.

Generally, although the number of publications utilizing machine learning tools for sensor development is considerably low, this approach has gained popularity recently, with the majority of the above-described works being published in 2025. As these reports have demonstrated, the implementation of ML strategies allows for enhanced sensitivity, fast and precise data analysis, and a decrease in the operations performed manually, i.e., the automatization of the detection process, which increases both the accuracy and the precision of the assays.

## Figures and Tables

**Table 5:** Pearson correlation coefficients for sensitivity and major QD parameters.

Parameter		Size			Excitation λ			Emission λ		
Type		FL	P	CL	FL	P	CL	FL	P	CL
Correl	LOD	0.384	0.181	0.181	0.115	−0.043	-	−0.227	−0.163	−0.210
	pLOD	−0.466	−0.299	−0.211	0.179	−0.126	-	0.313	0.133	−0.024
Sample Size		38	35	25	54	49	-	54	50	30

**Table 6:** Average and median values of essential QD parameters for FL, P, and CL (fluorescence, phosphorescence, and chemiluminescence).

Average	FL	P	CL	Median	FL	P	CL
pLOD	7.4	7.6	13.0	pLOD	7.6	7.4	13.6
Excitation λ	359	304	-	Excitation λ	360	299	-
Emission λ	509	590	602	Emission λ	530	590	615
Size	4.9	4.6	5.3	Size	3.3	3.8	4.8

## References

[1] Zhang Y., Liu B., Liu Z., Li J. (2022). Research progress in the synthesis and biological application of quantum dots. New J. Chem..

[2] Brichkin S.B., Razumov V.F. (2016). Colloidal quantum dots: Synthesis, properties and applications. Russ. Chem. Rev..

[3] Agarwal K., Rai H., Mondal S. (2023). Quantum dots: An overview of synthesis, properties, and applications. Mater. Res. Express.

[4] Valizadeh A., Mikaeili H., Samiei M., Farkhani S.M., Zarghami N., Kouhi M., Akbarzadeh A., Davaran S. (2012). Quantum dots: Synthesis, bioapplications, and toxicity. Nanoscale Res. Lett..

[5] Das A., Snee P.T. (2016). Synthetic Developments of Nontoxic Quantum Dots. ChemPhysChem.

[6] Bawendi M.G., Wilson W.L., Rothberg L., Carroll P.J., Jedju T.M., Steigerwald M.L., Brus L.E. (1990). Electronic structure and photoexcited-carrier dynamics in nanometer-size CdSe clusters. Phys. Rev. Lett..

[7] Klimov V.I. (2007). Spectral and Dynamical Properties of Multiexcitons in Semiconductor Nanocrystals. Annu. Rev. Phys. Chem..

[8] Steckel J.S., Snee P., Coe-Sullivan S., Zimmer J.P., Halpert J.E., Anikeeva P., Kim L.-A., Bulovic V., Bawendi M.G. (2006). Color-Saturated Green-Emitting QD-LEDs. Angew. Chem. Int. Ed..

[9] Jang E., Jun S., Pu L. (2003). High quality CdSeS nanocrystals synthesized by facile single injection process and their electroluminescence. Chem. Commun..

[10] Rempel A.A., Ovchinnikov O.V., Weinstein I.A., Rempel S.V., Kuznetsova Y.V., Naumov A.V., Smirnov M.S., Eremchev I.Y., Vokhmintsev A.S., Savchenko S.S. (2024). Quantum dots: Modern methods of synthesis and optical properties. Uspekhi Khimii.

[11] Ekimov A.I., Onushchenko A.A. (1981). Quantum size effect in three-dimensional microscopic semiconductor crystals. Lett. JETP.

[12] Reed M.A., Bate R.T., Bradshaw K., Duncan W.M., Frensley W.R., Lee J.W., Shih H.D. (1986). Spatial quantization in GaAs–AlGaAs multiple quantum dots. J. Vac. Sci. Technol. B Microelectron. Process. Phenom..

[13] Ekimov A.I., Onushchenko A.A. (1984). Size quantization of the electron energy spectrum in a microscopic semiconductor crystal. Lett. JETP.

[14] Ekimov A.I., Efros A.L., Onushchenko A.A. (1985). Quantum size effect in semiconductor microcrystals. Solid State Commun..

[15] Vandyshev Y.V., Dneprovskiǐ V.S., Ekimov A.I., Okorokov D.K., Popova L.B., Efros A.L. (1987). Nonlinear optical properties of semiconductor microcrystals. Sov. J. Exp. Theor. Phys. Lett..

[16] Ekimov A.I., Efros A.L., Ivanov M.G., Onushchenko A.A., Shumilov S.K. (1989). Donor-like exciton in zero-dimension semiconductor structures. Solid State Commun..

[17] Ekimov A.I., Efros A.L., Shubina T.V., Skvortsov A.P. (1990). Quantum-size stark effect in semiconductor microcrystals. J. Lumin..

[18] Ekimov A.I., Hache F., Schanne-Klein M.C., Ricard D., Flytzanis C., Kudryavtsev I.A., Yazeva T.V., Rodina A.V., Efros A.L. (1993). Absorption and intensity-dependent photoluminescence measurements on CdSe quantum dots: Assignment of the first electronic transitions. JOSA B.

[19] Itoh T., Nishijima M., Ekimov A.I., Gourdon C., Efros A.L., Rosen M. (1995). Polaron and exciton-phonon complexes in CuCl nanocrystals. Phys. Rev. Lett..

[20] Ekimov A. (1996). Growth and optical properties of semiconductor nanocrystals in a glass matrix. J. Lumin..

[21] Liu B., Altintas Y., Wang L., Shendre S., Sharma M., Sun H., Mutlugun E., Demir H.V. (2020). Record High External Quantum Efficiency of 19.2% Achieved in Light-Emitting Diodes of Colloidal Quantum Wells Enabled by Hot-Injection Shell Growth. Adv. Mater..

[22] Fang X., Han M., Lu G., Tu W., Dai Z. (2012). Electrochemiluminescence of CdSe quantum dots for highly sensitive competitive immunosensing. Sens. Actuators B Chem..

[23] Chen B., Liu J., Yang T., Chen L., Hou J., Feng C., Huang C.Z. (2019). Development of a portable device for Ag+ sensing using CdTe QDs as fluorescence probe via an electron transfer process. Talanta.

[24] Wang X., Liu Z., Gao P., Li Y., Qu X. (2020). Surface functionalized quantum dots as biosensor for highly selective and sensitive detection of ppb level of propafenone. Spectrochim. Acta Part A Mol. Biomol. Spectrosc..

[25] Yang W., Fidelis T.T., Sun W.-H. (2020). Machine Learning in Catalysis, From Proposal to Practicing. ACS Omega.

[26] Mater A.C., Coote M.L. (2019). Deep Learning in Chemistry. J. Chem. Inf. Model..

[27] Krishnadasan S., Brown R.J.C., deMello A.J., deMello J.C. (2007). Intelligent routes to the controlled synthesis of nanoparticles. Lab A Chip.

[28] Zhang Y., Ren D., Shi Y., Yuan R., Ye H., Yin X.-B., Chi H. (2025). A smartphone sensing fluorescent detection of mercury ion based on silicon quantum dots in environment water. Spectrochim. Acta Part A Mol. Biomol. Spectrosc..

[29] Ma Y., Mei H., Li Y., Zhou P., Mao G., Wang H., Wang X. (2022). A novel raiometric fluorescence probe based on silicon quantum dots and copper nanoclusters for visual assay of l-cysteine in milks. Food Chem..

[30] Wei N., Liang Z.-Y., Fang W.-L., Guo X.-F., Wang H., Zhang H.-X. (2023). Facile synthesis of non-modified yellow emission silicon quantum dots and their visualization of hydrogen sulfide in living cells and onion tissues. J. Colloid Interface Sci..

[31] Zhao D., Liu H., Zhang Z., Xiao X., Li J. (2022). Preparation of green luminescent silicon quantum dots by synergistic method for VB12 detection and antimicrobial property research application. Colloids Surf. B Biointerfaces.

[32] Wei N., Wei M.-X., Huang B.-H., Guo X.-F., Wang H. (2021). One-pot facile synthesis of green-emitting fluorescent silicon quantum dots for the highly selective and sensitive detection of nitrite in food samples. Dye. Pigment..

[33] Liu Y., Wang Q., Guo S., Jia P., Shui Y., Yao S., Huang C., Zhang M., Wang L. (2018). Highly selective and sensitive fluorescence detection of hydroquinone using novel silicon quantum dots. Sens. Actuators B Chem..

[34] Makwana K.P., Deshpande M.P., Malek N.I., Park T.J., Kailasa S.K. (2025). LaSrO3 perovskite quantum dots as a fluorescent probe for the detection of bilirubin and epinephrine via FRET and IFE mechanisms. J. Photochem. Photobiol. A Chem..

[35] Cernea M., Monnereau O., Llewellyn P., Tortet L., Galassi C. (2006). Sol–gel synthesis and characterization of Ce doped-BaTiO_3_. J. Eur. Ceram. Soc..

[36] Zhang Q., Jiang M., Yan G., Feng Y., Zhang B. (2022). Surface ligand engineering involving fluorophenethyl ammonium for stable and strong emission CsPbBr_3_ quantum dots and high-performance QLEDs. J. Mater. Chem. C.

[37] Jiang W., Kim B., Chae H. (2020). Phenethylamine ligand engineering of red InP quantum dots for improving the efficiency of quantum dot light-emitting diodes. Opt. Lett..

[38] Chen G., Zhu X., Xing C., Wang Y., Xu X., Bao J., Huang J., Zhao Y., Wang X., Zhou X. (2023). Machine Learning-Assisted Microfluidic Synthesis of Perovskite Quantum Dots. Adv. Photonics Res..

[39] Abdel-Latif K., Epps R.W., Bateni F., Han S., Reyes K.G., Abolhasani M. (2021). Self-Driven Multistep Quantum Dot Synthesis Enabled by Autonomous Robotic Experimentation in Flow. Adv. Intell. Syst..

[40] Atabaev T.S. (2018). Doped Carbon Dots for Sensing and Bioimaging Applications: A Minireview. Nanomaterials.

[41] Ding R., Chen Y., Wang Q., Wu Z., Zhang X., Li B., Lin L. (2022). Recent advances in quantum dots-based biosensors for antibiotics detection. J. Pharm. Anal..

[42] Sabzehmeidani M.M., Kazemzad M. (2022). Quantum dots based sensitive nanosensors for detection of antibiotics in natural products: A review. Sci. Total Environ..

[43] Mehta J., Dilbaghi N., Kumar Singhal N., Marrazza G., Kaushik A., Kumar S. (2023). Electrochemiluminescent quantum dots as emerging next generation sensing platforms. Chem. Eng. J..

[44] Sargazi S., Fatima I., Hassan Kiani M., Mohammadzadeh V., Arshad R., Bilal M., Rahdar A., Díez-Pascual A.M., Behzadmehr R. (2022). Fluorescent-based nanosensors for selective detection of a wide range of biological macromolecules: A comprehensive review. Int. J. Biol. Macromol..

[45] Wang Z., Yao B., Xiao Y., Tian X., Wang Y. (2023). Fluorescent Quantum Dots and Its Composites for Highly Sensitive Detection of Heavy Metal Ions and Pesticide Residues: A Review. Chemosensors.

[46] Sudewi S., Sai Sashank P.V., Kamaraj R., Zulfajri M., Huang G.G. (2024). Understanding Antibiotic Detection with Fluorescence Quantum Dots: A Review. J. Fluoresc..

[47] Zhong L., Zhang L., Li Y., Liang X., Kong L., Shen X., Wu T. (2021). Assessment of the Toxicity of Quantum Dots through Biliometric Analysis. Int. J. Environ. Res. Public Health.

[48] Bian F., Sun L., Cai L., Wang Y., Zhao Y. (2019). Quantum dots from microfluidics for nanomedical application. WIREs Nanomed. Nanobiotechnology.

[49] Sun B., Xie W., Yi G., Chen D., Zhou Y., Cheng J. (2001). Microminiaturized immunoassays using quantum dots as fluorescent label by laser confocal scanning fluorescence detection. J. Immunol. Methods.

[50] Sultangaziyev A., Bukasov R. (2020). Review: Applications of surface-enhanced fluorescence (SEF) spectroscopy in bio-detection and biosensing. Sens. Bio-Sens. Res..

[51] Sultangaziyev A., Akhmetova A., Kunushpayeva Z., Rapikov A., Filchakova O., Bukasov R. (2020). Aluminum foil as a substrate for metal enhanced fluorescence of bacteria labelled with quantum dots, shows very large enhancement and high contrast. Sens. Bio-Sens. Res..

[52] Bukasov R., Kunushpayeva Z., Rapikov A., Zhunussova S., Sultangaziyev A., Filchakova O. (2020). High Contrast Surface Enhanced Fluorescence of Carbon Dot Labeled Bacteria Cells on Aluminum Foil. J. Fluoresc..

[53] Bukasov R., Filchakova O., Gudun K., Bouhrara M. (2018). Strong Surface Enhanced Florescence of Carbon Dot Labeled Bacteria Cells Observed with High Contrast on Gold Film. J. Fluoresc..

[54] Qiushu C., Alper K., Xudong F. (2016). An Optofluidic FRET Laser Using Quantum Dots as Donor.

[55] Shopova S.I., Cupps J.M., Zhang P., Henderson E.P., Lacey S., Fan X. (2007). Opto-fluidic ring resonator lasers based on highly efficient resonant energy transfer. Opt. Express.

[56] Hong J., Pei D., Guo X. (2012). Quantum dot-Eu3+ conjugate as a luminescence turn-on sensor for ultrasensitive detection of nucleoside triphosphates. Talanta.

[57] Ban R., Zheng F., Zhang J. (2015). A highly sensitive fluorescence assay for 2,4,6-trinitrotoluene using amine-capped silicon quantum dots as a probe. Anal. Methods.

[58] Peveler W.J., Roldan A., Hollingsworth N., Porter M.J., Parkin I.P. (2016). Multichannel Detection and Differentiation of Explosives with a Quantum Dot Array. ACS Nano.

[59] Zhang Z., Li J., Wang X., Shen D., Chen L. (2015). Quantum Dots Based Mesoporous Structured Imprinting Microspheres for the Sensitive Fluorescent Detection of Phycocyanin. ACS Appl. Mater. Interfaces.

[60] Li Y., Xu J., Wang L., Huang Y., Guo J., Cao X., Shen F., Luo Y., Sun C. (2016). Aptamer-based fluorescent detection of bisphenol A using nonconjugated gold nanoparticles and CdTe quantum dots. Sens. Actuators B Chem..

[61] Qian J., Hua M., Wang C., Wang K., Liu Q., Hao N., Wang K. (2016). Fabrication of l-cysteine-capped CdTe quantum dots based ratiometric fluorescence nanosensor for onsite visual determination of trace TNT explosive. Anal. Chim. Acta.

[62] Singh K., Mehta S.K. (2016). Luminescent ZnO quantum dots as an efficient sensor for free chlorine detection in water. Analyst.

[63] Chang L., He X., Chen L., Zhang Y. (2017). Mercaptophenylboronic acid-capped Mn-doped ZnS quantum dots for highly selective and sensitive fluorescence detection of glycoproteins. Sens. Actuators B Chem..

[64] Qian J., Wang K., Wang C., Ren C., Liu Q., Hao N., Wang K. (2017). Ratiometric fluorescence nanosensor for selective and visual detection of cadmium ions using quencher displacement-induced fluorescence recovery of CdTe quantum dots-based hybrid probe. Sens. Actuators B Chem..

[65] Tang Z., Lin Z., Li G., Hu Y. (2017). Amino Nitrogen Quantum Dots-Based Nanoprobe for Fluorescence Detection and Imaging of Cysteine in Biological Samples. Anal. Chem..

[66] Yu M., Zhao K., Zhu X., Tang S., Nie Z., Huang Y., Zhao P., Yao S. (2017). Development of near-infrared ratiometric fluorescent probe based on cationic conjugated polymer and CdTe/CdS QDs for label-free determination of glucose in human body fluids. Biosens. Bioelectron..

[67] Zhou R., Zhao Q., Liu K.-K., Lu Y.-J., Dong L., Shan C.-X. (2017). Europium-decorated ZnO quantum dots as a fluorescent sensor for the detection of an anthrax biomarker. J. Mater. Chem. C.

[68] Pourghobadi Z., Mirahmadpour P., Zare H. (2018). Fluorescent biosensor for the selective determination of dopamine by TGA-capped CdTe quantum dots in human plasma samples. Opt. Mater..

[69] Xing X., Wang D., Chen Z., Zheng B., Li B., Wu D. (2018). ZnTe quantum dots as fluorescence sensors for the detection of iron ions. J. Mater. Sci. Mater. Electron..

[70] Zhao X., He D., Wang Y., Fu C. (2018). Facile fabrication of tungsten disulfide quantum dots (WS2 QDs) as effective probes for fluorescence detection of dopamine (DA). Mater. Chem. Phys..

[71] Feng J., Tao Y., Shen X., Jin H., Zhou T., Zhou Y., Hu L., Luo D., Mei S., Lee Y.-I. (2019). Highly sensitive and selective fluorescent sensor for tetrabromobisphenol-A in electronic waste samples using molecularly imprinted polymer coated quantum dots. Microchem. J..

[72] Li D., Xu X., Zhou P., Huang Y., Feng Y., Gu Y., Wang M., Liu Y. (2019). A facile synthesis of hybrid silicon quantum dots and fluorescent detection of bovine hemoglobin. New J. Chem..

[73] Najafi S., Safari M., Amani S., Mansouri K., Shahlaei M. (2019). Preparation, characterization and cell cytotoxicity of Pd-doped CdTe quantum dots and its application as a sensitive fluorescent nanoprobe. J. Mater. Sci. Mater. Electron..

[74] Safari M., Najafi S., Arkan E., Amani S., Shahlaei M. (2019). Facile aqueous synthesis of Ni-doped CdTe quantum dots as fluorescent probes for detecting pyrazinamide in plasma. Microchem. J..

[75] Wang Z., Xiao X., Zou T., Yang Y., Xing X., Zhao R., Wang Z., Wang Y. (2019). Citric Acid Capped CdS Quantum Dots for Fluorescence Detection of Copper Ions (II) in Aqueous Solution. Nanomaterials.

[76] Zhang Z., Ma X., Jia M., Li B., Rong J., Yang X. (2019). Deposition of CdTe quantum dots on microfluidic paper chips for rapid fluorescence detection of pesticide 2,4-D. Analyst.

[77] Zhang X., Li C., Zhao S., Pang H., Han Y., Luo X., Tang W., Li Z. (2020). S doped silicon quantum dots with high quantum yield as a fluorescent sensor for determination of Fe^3+^ in water. Opt. Mater..

[78] Gao R., Li D., Zhang Q., Zheng S., Ren X., Deng W. (2021). GNPs-QDs core–satellites assembly: Trimodal platform for on-site identification and detection of TNT in complex media. Sens. Actuators B Chem..

[79] Liu M., Bai Y., He Y., Zhou J., Ge Y., Zhou J., Song G. (2021). Facile microwave-assisted synthesis of Ti3C2 MXene quantum dots for ratiometric fluorescence detection of hypochlorite. Microchim. Acta.

[80] Yang M., Wang C., Liu E., Hu X., Hao H., Fan J. (2021). A novel ascorbic acid ratiometric fluorescent sensor based on ZnCdS quantum dots embedded molecularly imprinted polymer and silica-coated CdTeS quantum dots. J. Mol. Liq..

[81] Yi Y., Zeng W., Zhu G. (2021). β-Cyclodextrin functionalized molybdenum disulfide quantum dots as nanoprobe for sensitive fluorescent detection of parathion-methyl. Talanta.

[82] Zhang J., Wei Y., Qiu S., Xiong Y. (2021). A highly selective and simple fluorescent probe for salbutamol detection based on thioglycolic acid-capped CdTe quantum dots. Spectrochim. Acta Part A Mol. Biomol. Spectrosc..

[83] Zhao R., Wang Z., Tian X., Shu H., Yang Y., Xiao X., Wang Y. (2021). Excellent fluorescence detection of Cu^2+^ in water system using N-acetyl-L-cysteines modified CdS quantum dots as fluorescence probe. Nanotechnology.

[84] Aznar-Gadea E., Rodriguez-Canto P.J., Sánchez S.A., Martínez-Pastor J.P., Abargues R. (2022). Luminescent CdSe Quantum Dot Arrays for Rapid Sensing of Explosive Taggants. ACS Appl. Nano Mater..

[85] Wang X., Li L., Jiang H., Zhangsun H., Wang Q., Sun X., Wang L. (2022). Highly selective and sensitive fluorescence detection of tetracyclines based on novel tungsten oxide quantum dots. Food Chem..

[86] Narasimhappa P., Singh S., Ramamurthy P.C. (2023). Synthesis of water-soluble CdS quantum dots for the fluorescence detection of tetracycline. Environ. Pollut..

[87] Zhong Y., Guo L., Zou Y., Chen Y., Lu Z., Wang D. (2023). Rapid and ratiometric fluorescent detection of hypochlorite by glutathione functionalized molybdenum disulfide quantum dots. Spectrochim. Acta Part A Mol. Biomol. Spectrosc..

[88] Singh P.D.D., Murthy Z.V.P., Kailasa S.K. (2024). Zinc nitride quantum dots as an efficient probe for simultaneous fluorescence detection of Cu^2+^ and Mn^2+^ ions in water samples. Microchim. Acta.

[89] Velamala L.K., Patel M.R., Deshpande M.P., Gul A.R., Park T.J., Kailasa S.K. (2024). Fluorescence detection of superoxide anion with CsPbBr_3_ perovskite quantum dots in aqueous media. J. Mol. Liq..

[90] Zhang Y., Wang C., Wei G., Wang X., Liu W., Yang G., Zhang P., Li Q., Geng X., Chen L. (2024). Facile fluorescence detection of malachite green in fish using molecularly imprinted polymers doped CdTe quantum dots based system. Food Chem..

[91] Kailasa S.K., Patel M.R., Deshpande M.P., Shin E., Choi Y., Park T.J. (2025). Maltose-functionalized MAPbBr3 fluorescent perovskite quantum dots with strong water resistance for detection of γ-aminobutyric acid as a neurological biomarker. J. Photochem. Photobiol. A Chem..

[92] Zhang L., Shao K., Zhong Y., Guo L., Ge J., Lu Z., Wang D. (2025). Molybdenum disulfide quantum dots for rapid fluorescence detection of glutathione and ascorbic acid. Spectrochim. Acta Part A Mol. Biomol. Spectrosc..

[93] Creeden J.F., Gordon D.M., Stec D.E., Hinds T.D. (2020). Bilirubin as a metabolic hormone: The physiological relevance of low levels. Am. J. Physiol.-Endocrinol. Metab..

[94] Tzani M.A., Gioftsidou D.K., Kallitsakis M.G., Pliatsios N.V., Kalogiouri N.P., Angaridis P.A., Lykakis I.N., Terzidis M.A. (2021). Direct and Indirect Chemiluminescence: Reactions, Mechanisms and Challenges. Molecules.

[95] Han H., Sheng Z., Liang J. (2007). Electrogenerated chemiluminescence from thiol-capped CdTe quantum dots and its sensing application in aqueous solution. Anal. Chim. Acta.

[96] Lin Z., Xue W., Chen H., Lin J.-M. (2011). Peroxynitrous-Acid-Induced Chemiluminescence of Fluorescent Carbon Dots for Nitrite Sensing. Anal. Chem..

[97] Yang E., Zhang Y., Shen Y. (2022). Quantum dots for electrochemiluminescence bioanalysis—A review. Anal. Chim. Acta.

[98] Song H., Su Y., Zhang L., Lv Y. (2019). Quantum dots-based chemiluminescence probes: An overview. Luminescence.

[99] Yang G., He Y., Zhao J., Chen S., Yuan R. (2021). Ratiometric electrochemiluminescence biosensor based on Ir nanorods and CdS quantum dots for the detection of organophosphorus pesticides. Sens. Actuators B Chem..

[100] Li M., Li Z., Wang P., Ma Q. (2023). A novel bimetallic MXene derivative QD-based ECL sensor for miRNA-27a-3p detection. Biosens. Bioelectron..

[101] Li J., Wang Q., Xiong C., Deng Q., Zhang X., Wang S., Chen M.-M. (2022). An ultrasensitive CH_3_NH_3_PbBr_3_ quantum dots@SiO_2_-based electrochemiluminescence sensing platform using an organic electrolyte for aflatoxin B1 detection in corn oil. Food Chem..

[102] Wang J., Han H., Jiang X., Huang L., Chen L., Li N. (2012). Quantum Dot-Based Near-Infrared Electrochemiluminescent Immunosensor with Gold Nanoparticle-Graphene Nanosheet Hybrids and Silica Nanospheres Double-Assisted Signal Amplification. Anal. Chem..

[103] Liu S., Zhang X., Yu Y., Zou G. (2014). A Monochromatic Electrochemiluminescence Sensing Strategy for Dopamine with Dual-Stabilizers-Capped CdSe Quantum Dots as Emitters. Anal. Chem..

[104] Dong Y.-P., Gao T.-T., Zhou Y., Zhu J.-J. (2014). Electrogenerated Chemiluminescence Resonance Energy Transfer between Luminol and CdSe@ZnS Quantum Dots and Its Sensing Application in the Determination of Thrombin. Anal. Chem..

[105] Zhang J.-J., Kang T.-F., Hao Y.-C., Lu L.-P., Cheng S.-Y. (2015). Electrochemiluminescent immunosensor based on CdS quantum dots for ultrasensitive detection of microcystin-LR. Sens. Actuators B Chem..

[106] Wang J., Jiang X. (2015). Anodic near-infrared electrochemiluminescence from CdTe/CdS coresmall/shellthick quantum dots and their sensing ability of Cu^2+^. Sens. Actuators B Chem..

[107] Dong H., Han T.-T., Ren L.-L., Ding S.-N. (2017). Novel sandwich-structured electrochemiluminescence immunosensing platform via CdTe quantum dots-embedded mesoporous silica nanospheres as enhanced signal labels and Fe_3_O_4_@SiO_2_@PS nanocomposites as magnetic separable carriers. J. Electroanal. Chem..

[108] Zhao M., Chen A.-Y., Huang D., Chai Y.-Q., Zhuo Y., Yuan R. (2017). MoS2 Quantum Dots as New Electrochemiluminescence Emitters for Ultrasensitive Bioanalysis of Lipopolysaccharide. Anal. Chem..

[109] Wu F.-F., Zhou Y., Wang J.-X., Zhuo Y., Yuan R., Chai Y.-Q. (2017). A novel electrochemiluminescence immunosensor based on Mn doped Ag_2_S quantum dots probe for laminin detection. Sens. Actuators B Chem..

[110] Dong Y.-P., Wang J., Peng Y., Zhu J.-J. (2017). Electrogenerated chemiluminescence of Si quantum dots in neutral aqueous solution and its biosensing application. Biosens. Bioelectron..

[111] Yang X., Yu Y.-Q., Peng L.-Z., Lei Y.-M., Chai Y.-Q., Yuan R., Zhuo Y. (2018). Strong Electrochemiluminescence from MOF Accelerator Enriched Quantum Dots for Enhanced Sensing of Trace cTnI. Anal. Chem..

[112] Hu Y., Chen C., Liu Y., Wang S., Guo Z., Hu Y. (2018). Dual-signals electrochemiluminescence ratiometry based the synergic effect between luminol and CdSe quantum dots for direct detection of hydrogen peroxide. J. Electroanal. Chem..

[113] Zhang L., Tian K., Dong Y., Ding H., Wang C. (2018). Electrogenerated chemiluminescence of Ru(bpy)_3_^2+^ at a black phosphorus quantum dot modified electrode and its sensing application. Analyst.

[114] Wang Q., Li S., Li J. (2018). A molecularly imprinted sensor with enzymatic enhancement of electrochemiluminescence of quantum dots for ultratrace clopyralid determination. Anal. Bioanal. Chem..

[115] Lei Y.-M., Zhou J., Chai Y.-Q., Zhuo Y., Yuan R. (2018). SnS_2_ Quantum Dots as New Emitters with Strong Electrochemiluminescence for Ultrasensitive Antibody Detection. Anal. Chem..

[116] Gao J., Chen Z., Mao L., Zhang W., Wen W., Zhang X., Wang S. (2019). Electrochemiluminescent aptasensor based on resonance energy transfer system between CdTe quantum dots and cyanine dyes for the sensitive detection of Ochratoxin A. Talanta.

[117] Liu H., Zhang Y., Dong Y., Chu X. (2019). Electrogenerated chemiluminescence aptasensor for lysozyme based on copolymer nanospheres encapsulated black phosphorus quantum dots. Talanta.

[118] Du D., Shu J., Guo M., Haghighatbin M.A., Yang D., Bian Z., Cui H. (2020). Potential-Resolved Differential Electrochemiluminescence Immunosensor for Cardiac Troponin I Based on MOF-5-Wrapped CdS Quantum Dot Nanoluminophores. Anal. Chem..

[119] Chen P., Liu Z., Liu J., Liu H., Bian W., Tian D., Xia F., Zhou C. (2020). A novel electrochemiluminescence aptasensor based CdTe QDs@NH2-MIL-88(Fe) for signal amplification. Electrochim. Acta.

[120] Xu J., Zhang R., Liu C., Sun A., Chen J., Zhang Z., Shi X. (2020). Highly Selective Electrochemiluminescence Sensor Based on Molecularly Imprinted-quantum Dots for the Sensitive Detection of Cyfluthrin. Sensors.

[121] Liu Y., Li B., Yao Y., Yang B., Tian T., Miao Y., Liu B. (2021). An electrochemiluminescence sensor for 17β-estradiol detection based on resonance energy transfer in α-FeOOH@CdS/Ag NCs. Talanta.

[122] Feng D., Wei F., Wu Y., Tan X., Li F., Lu Y., Fan G., Han H. (2021). A novel signal amplified electrochemiluminescence biosensor based on MIL-53(Al)@CdS QDs and SiO2@AuNPs for trichlorfon detection. Analyst.

[123] Rasoulzadeh F., Amjadi M. (2021). The chemiluminescence of AgInS_2_ quantum dots and its application as a sensing platform for glutathione assay. J. Photochem. Photobiol. A Chem..

[124] Zhao J., He Y., Tan K., Yang J., Chen S., Yuan R. (2021). Novel Ratiometric Electrochemiluminescence Biosensor Based on BP-CdTe QDs with Dual Emission for Detecting MicroRNA-126. Anal. Chem..

[125] Jia M., Jia B., Liao X., Shi L., Zhang Z., Liu M., Zhou L., Li D., Kong W. (2022). A CdSe@CdS quantum dots based electrochemiluminescence aptasensor for sensitive detection of ochratoxin A. Chemosphere.

[126] Liu W., Su M., Chen A., Peng K., Chai Y., Yuan R. (2022). Highly Efficient Electrochemiluminescence Based on Luminol/MoS_2_ Quantum Dots@Zeolitic Imidazolate Framework-8 as an Emitter for Ultrasensitive Detection of MicroRNA. Anal. Chem..

[127] Yang F., Gong J., Li M., Jiang X., Zhang J., Liao M., Zhang H., Tremblay P.-L., Zhang T. (2023). Electrochemiluminescent CdS Quantum Dots Biosensor for Cancer Mutation Detection at Different Positions on Linear DNA Analytes. Anal. Chem..

[128] Liu M., Wang Y., Tang S., Wang W., Liang A., Luo A. (2024). A ratiometric molecular imprinted electrochemiluminescence sensor based on enhanced luminescence of CdSe@ZnS quantum dots by MXene@NaAsc for detecting uric acid. Bioelectrochemistry.

[129] Gong Q., Guo Y., Wang X., Zhang L., Liu D., Nie G. (2025). Dual quenching ECL strategy based on AgInZnS quantum dots and a new co-reaction promoter oxygen vacancy-modified P5AIn/TiO_2_ for sensitive CEA detection. Sens. Actuators B Chem..

[130] Chinnadayyala S.R., Park J., Le H.T.N., Santhosh M., Kadam A.N., Cho S. (2019). Recent advances in microfluidic paper-based electrochemiluminescence analytical devices for point-of-care testing applications. Biosens. Bioelectron..

[131] Veamatahau A., Jiang B., Seifert T., Makuta S., Latham K., Kanehara M., Teranishi T., Tachibana Y. (2015). Origin of surface trap states in CdS quantum dots: Relationship between size dependent photoluminescence and sulfur vacancy trap states. Phys. Chem. Chem. Phys..

[132] Wu P., He Y., Wang H.-F., Yan X.-P. (2010). Conjugation of Glucose Oxidase onto Mn-Doped ZnS Quantum Dots for Phosphorescent Sensing of Glucose in Biological Fluids. Anal. Chem..

[133] Chang N., Mao J., Lu Y., Yang J., Pu Y., Zhang S., Liu Y. (2016). Time-resolved phosphorescent sensor array based on quantum dots for recognition of proteins. Sens. Actuators B Chem..

[134] Li D., Qin J., Lv J., Yang J., Yan G. (2018). “Turn on” room-temperature phosphorescent biosensors for detection of hyaluronic acid based on manganese-doped ZnS quantum dots. RSC Adv..

[135] Wang Y.-Q., Zou W.-S. (2011). 3-Aminopropyltriethoxysilane-functionalized manganese doped ZnS quantum dots for room-temperature phosphorescence sensing ultratrace 2,4,6-trinitrotoluene in aqueous solution. Talanta.

[136] He Y., Wang H.-F., Yan X.-P. (2008). Exploring Mn-Doped ZnS Quantum Dots for the Room-Temperature Phosphorescence Detection of Enoxacin in Biological Fluids. Anal. Chem..

[137] Miao Y., Wang R., Suna X., Yan G. (2019). Preparation of DNA functional phosphorescent quantum dots and application in melamine detection in milk. RSC Adv..

[138] Wu P., Zhao T., Tian Y., Wu L., Hou X. (2013). Protein-Directed Synthesis of Mn-Doped ZnS Quantum Dots: A Dual-Channel Biosensor for Two Proteins. Chemistry.

[139] Fan M., Gan T., Yin G., Cheng F., Zhao N. (2021). Molecularly imprinted polymer coated Mn-doped ZnS quantum dots embedded in a metal–organic framework as a probe for selective room temperature phosphorescence detection of chlorpyrifos. RSC Adv..

[140] Verma N., Singh A.K., Saini N. (2017). Synthesis and characterization of ZnS quantum dots and application for development of arginine biosensor. Sens. Bio-Sens. Res..

[141] Wang H.F., Li Y., Wu Y.Y., He Y., Yan X.P. (2010). Ascorbic acid induced enhancement of room temperature phosphorescence of sodium tripolyphosphate-capped Mn-Doped ZnS quantum dots: Mechanism and bioprobe applications. Chemistry.

[142] He Y., Yan X. (2011). Mn-doped ZnS quantum dots/methyl violet nanohybrids for room temperature phosphorescence sensing of DNA. Sci. China Chem..

[143] Zhang L., Zhang R., Cui P., Cao W., Gao F. (2013). An efficient phosphorescence energy transfer between quantum dots and carbon nanotubes for ultrasensitive turn-on detection of DNA. Chem. Commun..

[144] Dan L., Wang H.-F. (2013). Mn-Doped ZnS Quantum Dot Imbedded Two-Fragment Imprinting Silica for Enhanced Room Temperature Phosphorescence Probing of Domoic Acid. Anal. Chem..

[145] Tan L., Kang C., Xu S., Tang Y. (2013). Selective room temperature phosphorescence sensing of target protein using Mn-doped ZnS QDs-embedded molecularly imprinted polymer. Biosens. Bioelectron..

[146] Wang H.-F., Wu Y.-Y., Yan X.-P. (2013). Room-Temperature Phosphorescent Discrimination of Catechol from Resorcinol and Hydroquinone Based on Sodium Tripolyphosphate Capped Mn-Doped ZnS Quantum Dots. Anal. Chem..

[147] Bian W., Ma J., Guo W., Lu D., Fan M., Wei Y., Li Y., Shuang S., Choi M.M.F. (2013). Phosphorescence detection of L-ascorbic acid with surface-attached N-acetyl-L-cysteine and L-cysteine Mn doped ZnS quantum dots. Talanta.

[148] Bian W., Ma J., Liu Q., Wei Y., Li Y., Dong C., Shuang S. (2014). A novel phosphorescence sensor for Co^2+^ ion based on Mn-doped ZnS quantum dots. Luminescence.

[149] Zhu D., Li W., Wen H.-M., Chen Q., Ma L., Hu Y. (2014). Microwave-assisted aqueous synthesis of Mn-doped ZnS quantum dots and their room-temperature phosphorescence detection of indapamide. Anal. Methods.

[150] Gong Y., Fan Z. (2014). Melamine-modulated mercaptopropionic acid-capped manganese doped zinc sulfide quantum dots as a room-temperature phosphorescence sensor for detecting clenbuterol in biological fluids. Sens. Actuators B Chem..

[151] Zhang Z., Miao Y., Zhang Q., Yan G. (2015). Facile and sensitive detection of protamine by enhanced room-temperature phosphorescence of Mn-doped ZnS quantum dots. Anal. Biochem..

[152] Bi L., Yu Y.-H. (2015). Phosphorescent quantum dots/ethidium bromide nanohybrids based on photoinduced electron transfer for DNA detection. Spectrochim. Acta Part A Mol. Biomol. Spectrosc..

[153] Gong Y., Fan Z. (2015). Highly selective manganese-doped zinc sulfide quantum dots based label free phosphorescent sensor for phosphopeptides in presence of zirconium (IV). Biosens. Bioelectron..

[154] Zhang Z., Miao Y., Lian L., Yan G. (2015). Detection of quercetin based on Al^3+^-amplified phosphorescence signals of manganese-doped ZnS quantum dots. Anal. Biochem..

[155] Ertas N., Satana Kara H.E. (2015). l-Cysteine capped Mn-doped ZnS quantum dots as a room temperature phosphorescence sensor for in-vitro binding assay of idarubicin and DNA. Biosens. Bioelectron..

[156] Bian W., Wang F., Wei Y., Wang L., Liu Q., Dong W., Shuang S., Choi M.M.F. (2015). Doped zinc sulfide quantum dots based phosphorescence turn-off/on probe for detecting histidine in biological fluid. Anal. Chim. Acta.

[157] Zhang J., Zhu A., Zhao T., Wu L., Wu P., Hou X. (2015). Glucose oxidase-directed, instant synthesis of Mn-doped ZnS quantum dots in neutral media with retained enzymatic activity: Mechanistic study and biosensing application. J. Mater. Chem. B.

[158] Chen J., Zhu Y., Zhang Y. (2016). Glutathione-capped Mn-doped ZnS quantum dots as a room-temperature phosphorescence sensor for the detection of Pb^2+^ ions. Spectrochim. Acta Part A Mol. Biomol. Spectrosc..

[159] Gong Y., Fan Z. (2016). Room-Temperature Phosphorescence Turn-on Detection of DNA Based on Riboflavin-Modulated Manganese Doped Zinc Sulfide Quantum Dots. J. Fluoresc..

[160] Liu W., Li H., Wei Y., Dong C. (2017). A label-free phosphorescence sensing platform for trypsin based on Mn-ZnS QDs. RSC Adv..

[161] Lv J., Miao Y., Yan G. (2017). Detection of tumor marker miRNA21 based on phosphorescent resonance energy transfer of Mn–ZnS QDs. RSC Adv..

[162] Zhang J., Lu X., Lei Y., Hou X., Wu P. (2017). Exploring the tunable excitation of QDs to maximize the overlap with the absorber for inner filter effect-based phosphorescence sensing of alkaline phosphatase. Nanoscale.

[163] Zhang C., Zhang K., Zhao T., Liu B., Wang Z., Zhang Z. (2017). Selective phosphorescence sensing of pesticide based on the inhibition of silver(I) quenched ZnS:Mn^2+^ quantum dots. Sens. Actuators B Chem..

[164] Pacheco M.E., Castells C.B., Bruzzone L. (2017). Mn-doped ZnS phosphorescent quantum dots: Coumarins optical sensors. Sens. Actuators B Chem..

[165] Zhang W., Han Y., Chen X., Luo X., Wang J., Yue T., Li Z. (2017). Surface molecularly imprinted polymer capped Mn-doped ZnS quantum dots as a phosphorescent nanosensor for detecting patulin in apple juice. Food Chem..

[166] Deng P., Lu L.-Q., Cao W.-C., Tian X.-K. (2017). Phosphorescence detection of manganese(VII) based on Mn-doped ZnS quantum dots. Spectrochim. Acta Part A Mol. Biomol. Spectrosc..

[167] Lv J., Miao Y., Yang J., Qin J., Li D., Yan G. (2017). A DNA probe based on phosphorescent resonance energy transfer for detection of transgenic 35S promoter DNA. Biosens. Bioelectron..

[168] Li D., Qin J., Yan G. (2018). A phosphorescent sensor for detection of Micrococcal nuclease base on phosphorescent resonance energy transfer between quantum dots and DNA-ROX. Sens. Actuators B Chem..

[169] Wei X., Yu M., Li C., Gong X., Qin F., Wang Z. (2018). Magnetic nanoparticles coated with a molecularly imprinted polymer doped with manganese-doped ZnS quantum dots for the determination of 2,4,6-trichlorophenol. Microchim. Acta.

[170] Liu C.L., Hou C.J., Huo D.Q. (2018). Enhanced Room-Temperature Phosphorescence of Mn-Doped ZnS Quantum Dots Composited with PDDA for Detection of Adriamycin. J. Nanosci. Nanotechnol..

[171] Zou W.S., Deng M.Y., Wang Y.Q., Zhao X., Li W.H., Huang X.H. (2018). Alginate capped and manganese doped ZnS quantum dots as a phosphorescent probe for time-resolved detection of copper(II). Mikrochim. Acta.

[172] Qin J., Zheng J., Fang X., Yan G. (2018). Detection of resveratrol by phosphorescence quantum dots without conjunction and mutual impact exploration. RSC Adv..

[173] Luo S., Miao Y., Guo J., Sun X., Yan G. (2019). Phosphorimetric determination of 4-nitrophenol using mesoporous molecular imprinting polymers containing manganese(II)-doped ZnS quantum dots. Mikrochim. Acta.

[174] Chen S., Li Y., Wu S., Jiang X., Yang H., Su X., He L., Zou L., Ao X., Liu S. (2019). A phosphorescent probe for cephalexin consisting of mesoporous thioglycolic acid-modified Mn:ZnS quantum dots coated with a molecularly imprinted polymer. Mikrochim. Acta.

[175] Zhao J., Fan Z. (2019). Aggregation-induced phosphorescence quenching method for the detection of picric acid based on melamine-passivated Mn-doped ZnS quantum dots. Spectrochim. Acta Part A Mol. Biomol. Spectrosc..

[176] Liu Z., Hou J., He Q., Luo X., Huo D., Hou C. (2020). New application of Mn-doped ZnS quantum dots: Phosphorescent sensor for the rapid screening of chloramphenicol and tetracycline residues. Anal. Methods.

[177] Madurangika Jayasinghe G.D.T., Domínguez-González R., Bermejo-Barrera P., Moreda-Piñeiro A. (2020). Room temperature phosphorescent determination of aflatoxins in fish feed based on molecularly imprinted polymer—Mn-doped ZnS quantum dots. Anal. Chim. Acta.

[178] Jinadasa K.K., Peña-Vázquez E., Bermejo-Barrera P., Moreda-Piñeiro A. (2020). Synthesis and application of a surface ionic imprinting polymer on silica-coated Mn-doped ZnS quantum dots as a chemosensor for the selective quantification of inorganic arsenic in fish. Anal. Bioanal. Chem..

[179] Lv X., Gao P. (2020). An optical sensor for selective detection of phenol via double cross-linker precipitation polymerization. RSC Adv..

[180] Liu S., Li D., Shi D., Zhang G., Luo X., Xu Q., Zhao L., Guo J., Yan G. (2021). Construction of a room-temperature phosphorescent quantum dot probe and quantitative detection of thyroxine and carbamazepine. J. Mol. Struct..

[181] Qin G., Zuo L., Wei Y., Wang L., Bodwell G. (2021). Highly sensitive detection for alkaline phosphatase using doped ZnS quantum dots with room temperature phosphorescence and its logic gate function. Colloids Surf. B Biointerfaces.

[182] Chen S., Su X., Yuan C., Jia C.Q., Qiao Y., Li Y., He L., Zou L., Ao X., Liu A. (2021). A magnetic phosphorescence molecularly imprinted polymers probe based on manganese-doped ZnS quantum dots for rapid detection of trace norfloxacin residual in food. Spectrochim. Acta Part A Mol. Biomol. Spectrosc..

[183] Kong W., Liu M., Zhang J., Wu H., Wang Y., Su Q., Li Q., Zhang J., Wu C., Zou W.-S. (2023). Room-temperature phosphorescence and fluorescence nanocomposites as a ratiometric chemosensor for high-contrast and selective detection of 2,4,6-trinitrotoluene. Anal. Chim. Acta.

[184] Yang J., Zhe L., Bo Y., Xiangdong G., Feiyong Q., Yanping M., Dehui L., Hongmei G., Li W. (2025). Determination of Lead (II) by Phosphorescence Using Manganese-Doped Zinc Sulfide Quantum Dots. Anal. Lett..

[185] Forcada S., Sánchez-Visedo A., Melendreras C., Menéndez-Miranda M., Costa-Fernández J.M., Royo L.J., Soldado A. (2022). Design and Evaluation of a Competitive Phosphorescent Immunosensor for Aflatoxin M1 Quantification in Milk Samples Using Mn:ZnS Quantum Dots as Antibody Tags. Chemosensors.

[186] Hua L., Han H., Zhang X. (2009). Size-dependent electrochemiluminescence behavior of water-soluble CdTe quantum dots and selective sensing of l-cysteine. Talanta.

[187] Nakayama M., Kitano T., Ye J., Jin J. (2020). Anodic Electrochemiluminescence of CdTe Quantum Dots Using Tripropylamine as Coreactant: Size-dependent Effect. Anal. Sci..

[188] Zhang Y., Clapp A. (2011). Overview of Stabilizing Ligands for Biocompatible Quantum Dot Nanocrystals. Sensors.

[189] Wang S., Du L., Jin Z., Xin Y., Mattoussi H. (2020). Enhanced Stabilization and Easy Phase Transfer of CsPbBr_3_ Perovskite Quantum Dots Promoted by High-Affinity Polyzwitterionic Ligands. J. Am. Chem. Soc..

[190] Kovalenko M.V., Protesescu L., Bodnarchuk M.I. (2017). Properties and potential optoelectronic applications of lead halide perovskite nanocrystals. Science.

[191] Sanjayan C.G., Jyothiab M.S., Balakrishna R.G. (2022). Stabilization of CsPbBr3 quantum dots for photocatalysis, imaging and optical sensing in water and biological medium: A review. J. Mater. Chem. C.

[192] Yu M., Yang J., Zhang X., Yuan M., Zhang J., Gao L., Tang J., Lan X. (2024). In-Synthesis Se-Stabilization Enables Defect and Doping Engineering of HgTe Colloidal Quantum Dots. Adv. Mater..

[193] Parani S., Pandian K., Oluwafemi O.S. (2018). Gelatin stabilization of quantum dots for improved stability and biocompatibility. Int. J. Biol. Macromol..

[194] Wang M., Zhang M., Qian J., Zhao F., Shen L., Scholes G.D., Winnik M.A. (2009). Enhancing the Photoluminescence of Polymer-Stabilized CdSe/CdS/ZnS Core/Shell/Shell and CdSe/ZnS Core/Shell Quantum Dots in Water through a Chemical-Activation Approach. Langmuir.

[195] Nie Q., Tan W.B., Zhang Y. (2006). Synthesis and characterization of monodisperse chitosan nanoparticles with embedded quantum dots. Nanotechnology.

[196] Ali M., Zayed D., Ramadan W., Kamel O.A., Shehab M., Ebrahim S. (2019). Synthesis, characterization and cytotoxicity of polyethylene glycol-encapsulated CdTe quantum dots. Int. Nano Lett..

[197] Hens B., Smothers J., Rizvanovic H., Patel R., Wu Q., Kim K. (2020). The Future of Anticancer Drugs: A Cytotoxicity Assessment Study of CdSe/ZnS Quantum Dots. J. Nanotheranostics.

[198] Cheng Q., Duan Y., Fan W., Li D., Zhu C., Ma T., Liu J., Yu M. (2023). Cellular uptake, intracellular behavior, and acute/sub-acute cytotoxicity of a PEG-modified quantum dot with promising in-vivo biomedical applications. Heliyon.

[199] Zhang J., Zhou W., Qi H., He X. (2025). Deep-Learning-Assisted Digital Fluorescence Immunoassay on Magnetic Beads for Ultrasensitive Determination of Protein Biomarkers. Anal. Chem..

[200] Saren G., Zhu L., Han Y. (2022). Quantitative Detection of Gastrointestinal Tumor Markers Using a Machine Learning Algorithm and Multicolor Quantum Dot Biosensor. Comput. Intell. Neurosci..

[201] Zhang S., Zhu W., Zhang X., Mei L., Liu J., Wang F. (2025). Machine learning-driven fluorescent sensor array using aqueous CsPbBr_3_ perovskite quantum dots for rapid detection and sterilization of foodborne pathogens. J. Hazard. Mater..

